# Alix Serves as an Adaptor That Allows Human Parainfluenza Virus Type 1 to Interact with the Host Cell ESCRT System

**DOI:** 10.1371/journal.pone.0059462

**Published:** 2013-03-19

**Authors:** Jim Boonyaratanakornkit, Henrick Schomacker, Peter Collins, Alexander Schmidt

**Affiliations:** Laboratory of Infectious Diseases, RNA Viruses Section, National Institute of Allergy and Infectious Diseases, National Institutes of Health, Bethesda, Maryland, United States of America; Nanyang Technological University, Singapore

## Abstract

The cellular ESCRT (endosomal sorting complex required for transport) system functions in cargo-sorting, in the formation of intraluminal vesicles that comprise multivesicular bodies (MVB), and in cytokinesis, and this system can be hijacked by a number of enveloped viruses to promote budding. The respiratory pathogen human parainfluenza virus type I (HPIV1) encodes a nested set of accessory C proteins that play important roles in down-regulating viral transcription and replication, in suppressing the type I interferon (IFN) response, and in suppressing apoptosis. Deletion or mutation of the C proteins attenuates HPIV1 *in vivo*, and such mutants are being evaluated preclinically and clinically as vaccines. We show here that the C proteins interact and co-localize with the cellular protein Alix, which is a member of the class E vacuolar protein sorting (Vps) proteins that assemble at endosomal membranes into ESCRT complexes. The HPIV1 C proteins interact with the Bro1 domain of Alix at a site that is also required for the interaction between Alix and Chmp4b, a subunit of ESCRT-III. The C proteins are ubiquitinated and subjected to proteasome-mediated degradation, but the interaction with Alix_Bro1_ protects the C proteins from degradation. Neither over-expression nor knock-down of Alix expression had an effect on HPIV1 replication, although this might be due to the large redundancy of Alix-like proteins. In contrast, knocking down the expression of Chmp4 led to an approximately 100-fold reduction in viral titer during infection with wild-type (WT) HPIV1. This level of reduction was similar to that observed for the viral mutant, P(C-) HPIV1, in which expression of the C proteins were knocked out. Chmp4 is capable of out-competing the HPIV1 C proteins for binding Alix. Together, this suggests a possible model in which Chmp4, through Alix, recruits the C proteins to a common site on intracellular membranes and facilitates budding.

## Introduction

Human parainfluenza virus type 1 (HPIV1) is an important respiratory pathogen that causes a significant burden of disease, mainly in young children, the immunocompromised, and the elderly [1], [2], [3], [4], [5], [6], [7]. In children, HPIV1 is recognized as the most common cause of laryngotracheobronchitis or croup, a disease characterized by inspiratory stridor, a barking cough, and hoarseness [7]. HPIV1 is a single stranded, negative sense, non-segmented RNA virus in the Paramyxoviridae family. The viral genome is 15,600 nucleotides in length and contains 6 genes (3′ – N-P/C-M-F-HN-L –5′) that encode the nucleoprotein (N), phosphoprotein (P), C proteins (C), matrix protein (M), fusion protein (F), hemagglutinin-neuraminidase (HN), and the large polymerase (L). Each gene encodes a single major protein with the exception of the P/C gene, which encodes the structural P protein in one open reading frame and a nested set of four accessory carboxy-coterminal C proteins (C’, C, Y1, and Y2, in order of decreasing size) expressed from individual start sites in a second open reading frame. The use of an alternative and less efficient GTG start codon for translation of the C’ protein allows a proportion of ribosomes to scan, bypass, and initiate at the downstream start codons for translation of the P protein and of the shorter C protein isoforms.

The C proteins play a critical role in HPIV1 virulence by inhibiting apoptosis, regulating type I IFN production and signaling, and affecting the transcription of a large number of host genes [8], [9], [10], [11]. The C proteins of Sendai virus (SeV, murine PIV1), and HPIV1 share considerable sequence homology. However, important differences exist between the two viruses including differences in the immune evasion proteins and their activities [8], [12]. The P/C gene organization of SeV differs from that of HPIV1 in that SeV expresses, in addition to the C proteins, a second accessory protein, the V protein, that also exerts an inhibitory role on the host innate antiviral response [13]. For example, the V protein interacts with the cytosolic double-stranded (ds) RNA sensor MDA5 in order to block IFN production [11], [13], [14]. HPIV1 does not express a V protein, and its C proteins are the only known HPIV1 antagonists of the innate immune response. We previously reported that the induction of IFN**β** in response to HPIV1 infection relied mainly on MDA5 and is inhibited by the C proteins. However, we were unable to find any evidence that the HPIV1 C proteins directly interfered with any of the steps in the signal transduction pathway leading to IFN**β** production [14]. Instead, the effect appeared to be indirect: the C proteins were found to down-regulate viral RNA synthesis and limit the formation of cytoplasmic dsRNA that would otherwise activate MDA5 and PKR [14]. SeV and HPIV1 also differ in the way they interfere with type I IFN signaling. The mechanism by which the SeV C proteins accomplish this remains controversial and may involve the inhibition of STAT2 phosphorylation or the degradation of STAT1 [15], [16], whereas the HPIV1 C proteins do not facilitate degradation of the STAT proteins nor do they block their phosphorylation. Instead, the HPIV1 C proteins bind to STAT1 and sequester it in cytoplasmic aggregates located in the vicinity of the late endosomes, thereby preventing nuclear translocation and the subsequent activation of IFN-stimulated response elements [17].

In the present study, we extended our search for cellular proteins that might interact with the HPIV1 C proteins identified binding with the cellular protein Alix (apoptosis-linked gene-2-interacting protein X). Alix is one of a number of human proteins that act as adapters in the ESCRT pathway and contain a so-called Bro1 domain (a boomerang-shaped domain that is involved in protein-protein interactions and that localizes proteins to the late endosome). The ESCRT pathway involves more than 30 proteins that form complexes called ESCRT-0, -I, -II, and –III that act sequentially to sequester ubiquitinated protein cargo into vesicles that bud inwardly into endosomes and form multivesicular bodies (MVBs). Alix is a cytoplasmic protein comprised of 3 domains: Bro1, V, and a proline-rich region (PRR). Alix is an adaptor for several ESCRT-III subunits, including several isoforms of Chmp4 (charged multivesicular body protein), and has a myriad of roles in membrane biology, including cytokinesis, membrane abscission, receptor trafficking, signaling, and apoptosis. A number of enveloped viruses have so-called late domains in Gag or matrix proteins that hijack the ESCRT system to promote viral budding [18], [19], [20]. For example, for many of the retroviruses, the viral Gag proteins contain YP(X_n_)L sequence motifs, where n = 1−3, that bind a hydrophobic pocket on the 2^nd^ arm of the V domain of Alix. This interaction is required for budding through the MVB and plasma membrane [21], [22]. Alix has also been proposed to facilitate the release of SeV through the viral M and C proteins, although this finding remains controversial [23], [24], [25], [26].

Alix can recruit Chmp4 and other ESCRT-III subunits to various membranes during retroviral budding and cell division, including the plasma membrane and the midbody [19], [21], [22]. Conversely, the membrane bound Chmp4 can recruit Alix to late endosomes via an interaction with the Bro1 domain so that Alix can participate in MVB formation [27]. The Bro1 domain contains a conserved and exposed hydrophobic patch, and mutations in this patch, in particular the I212D substitution, inhibit the interaction between Chmp4 and Bro1, interfere with the recruitment of Bro1 to endosomes, block protein sorting in the MVB pathway, and inhibit HIV budding and release [21], [27].

We have generated a number of HPIV1 mutants to investigate C protein function, as well as to generate live vaccines that are attenuated *in vivo* by disturbing the IFN antagonist function of the C proteins [9], [28], [29]. A phenylalanine to serine substitution of amino acid 170 (C^F170S^) was introduced into the C protein of cDNA-derived HPIV1 (F170S HPIV1) by reverse genetics. This mutation permitted IFN**β** production during infection and resulted in restricted replication in the respiratory tract of hamsters and African green monkeys (AGMs) [9], [28], [30], [31]. A stabilized version of this mutation is present in a live-attenuated investigational HPIV1 vaccine that is currently in Phase 1 trials in children (ClinicalTrials.gov ID: NCT00641017). Another virus, referred to as P(C-), does not express any of the four C proteins due to point mutations that silence the C ORF *in vivo* [11]. We have found that this virus is severely impaired in its ability to replicate and spread. In the present study, we set out to define the interaction between the HPIV1 C proteins and Alix and the role of this interaction during an active infection. Here, we show that the viral C proteins and Chmp4 share a common binding site in the Bro1 domain of Alix and that this interaction is important in determining the intracellular localization of the C proteins, protecting the C proteins from proteasome-mediated degradation, and facilitating viral replication and spread.

## Methods

### Cells and Viruses

Human respiratory epithelial A549 cells and human embryonic kidney 293 T cells were maintained in Dulbecco’s modified Eagle’s medium (DMEM) supplemented with 10% fetal bovine serum (FBS) and 0.1 mg/mL gentamicin sulfate. Recombinant wild-type (WT) HPIV1 and P(C-) HPIV1 were propagated on MK2 cells (ATCC CCL7.1) in the presence of 1.2% trypsin TrypLE Select (Invitrogen) and purified on sucrose-step-gradients as previously described [10]. Viral titers were determined by a limiting dilution assay on MK2 cells in the presence of trypsin using hemadsorption to visualize infected wells [10]. All of the experiments involving HPIV1 infection employed a multiplicity of infection (MOI) of five 50% tissue culture infective doses (TCID_50_)/ml unless otherwise indicated. Infected cells were incubated at 37°C in the presence of trypsin and were analyzed from 24 to 120 h post-infection.

### Plasmid Constructs

**Figure 1:**
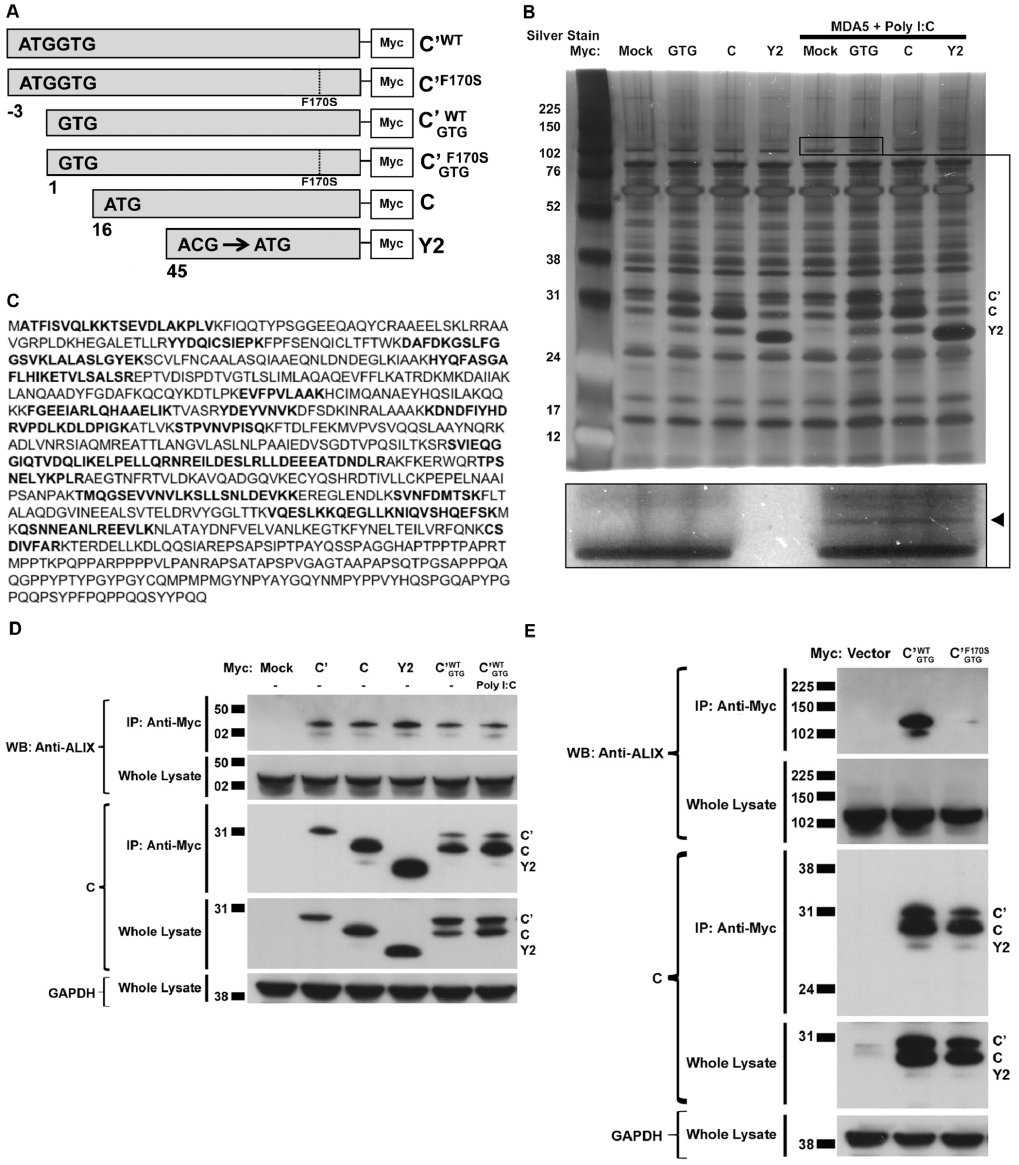
The cellular protein Alix interacts with the HPIV1 C proteins. **A.** Schematic diagram of HPIV1 C expression constructs with a Myc tag attached at the c-terminus. The cDNAs were constructed to contain the coding sequence for C’, C, and Y2 (the cDNA for the Y1 coding sequence was also constructed but not used in this study). The numbers denote the nucleotide position of the start codon relative to the start codon for C’. The location of the F170S point mutation is indicated by the dotted line. The C’^WT^_GTG_ and C’^F170S^_GTG_ constructs bear the native GTG codon as the translational start codon for C’. In contrast, for constructs C’^WT^ and C’^F170S^, an ATG start codon was placed immediately before the native GTG start codon to increase the expression of the C’ isoform at the expense of the smaller C isoforms. The native ACG start codon in the Y2 construct was replaced with an ATG codon to increase the expression of the Y2 isoform. **B.** Detection of a cellular species that co-immunoprecipitated with the HPIV1 C proteins. The above Myc-tagged C expression constructs were transfected into duplicate cultures of 293 T cells. One set was also transfected with MDA5 and, 42 h post-transfection, transfected again with poly(I:C). 48 h after the initial transfection, both sets of cultures were lysed and subjected to immunoprecipitation with anti-Myc antibodies to isolate the C proteins and possible binding partners, and the precipitated proteins were separated by SDS-PAGE and visualized by silver staining. A portion of the gel is magnified in the inset. The arrow indicates a representative band that co-immunoprecipitated with the Myc-tagged C proteins, regardless of transfection with MDA5 and poly(I:C). This band was excised from a duplicate gel that had been run in parallel and stained with Coomassie blue, and the band was analyzed by mass spectrometry. **C.** Identification of the co-immunoprecipitated species as the cellular protein Alix. The amino acid sequence of human Alix protein is shown, and peptide sequences that were identified by mass spectrometry are indicated in bold. **D.** Testing the interaction between endogenous Alix and the HPIV1 C’, C, and Y2 proteins. 293 T cells were transfected with plasmids encoding the indicated Myc-labeled C proteins. Similar to that described in panel B, a subset of cells were also co-transfected with MDA5 and, 42 h post-transfection, transfected again with poly(I:C). The cells were lysed and analyzed by immunoprecipitation with anti-Myc antibodies to isolate C proteins followed by SDS-PAGE and Western blot analysis using an Alix-specific antibody. Endogenous Alix co-immunoprecipitated with the WT C’, C, and Y2 proteins regardless of the presence of MDA5 and poly (I:C). **E.** Testing the interaction between endogenous Alix and the HPIV1 F170S mutation-containing C protein. As described in panel D, cellular lysates were immunoprecipitated with anti-Myc antibodies to isolate the C proteins. Endogenous Alix co-immunoprecipitated with the WT C proteins but not with the F170S mutation-containing C proteins.

Expression constructs were designed to express the C’, C, and Y2 isoforms, each containing a C-terminal Myc tag (**Fig. 1A**). In the C’^WT^ and C’^F170S^ constructs, an ATG start codon was inserted upstream of the native GTG start codon to increase the strength of the C’ start sequence, thereby increasing the expression of C’ at the expense of the downstream isoforms (C, Y1, and Y2). In the C’^WT^_GTG_ and the C’^F170S^_GTG_ constructs, the native GTG start codon was maintained. Constructs were also designed to individually express the C and Y2 isoforms. For the Y2 construct, the start codon was modified from the native ACG to an ATG start codon to increase Y2 protein expression. These cDNA were generated by PCR of the full-length antigenomic cDNA for WT HPIV1 and F170S HPIV1 and constructed with the insertion of a C-terminal c-Myc tag (amino acid sequence EQKLISEEDL) and flanking KpnI and EagI restriction sequences to allow cloning into the expression plasmid pcDNA3.1 (Invitrogen). Forward primers: C’^WT^ and C’^F170S^ – CATCGGTACCATGGTGGATACCTCAGCATCCAAAACTCTCC; C’^WT^_GTG_ and C’^F170S^_GTG_ – CATCGGTACCGTGGATACCTCAGCATCCAAAACTCTCCTTCCC; C –CATCGGTACCATGCCTTCTTTTTTGAGAGGGATCCTGAAGCC; Y1– CATCGGTACCATGTCATCGGACTCCTTGACGTCGTC; Y2–CATCGGTACCATGACGTCGTCTTATCCTACAAGCCCAC. Reverse primer: ATCACATGCGGCCGCTTACAGATCTTCTTCAGAAATAAGTTTTTGTTCTTCTTGTACTATGTGTGCTGCTAGTTTCC.

The HA-Alix construct, which expresses human Alix protein with a N-terminal HA tag from the plasmid pHCMV, was kindly provided by James Hurley (National Institute of Diabetes and Digestive and Kidney Diseases, NIH) [18], [32]. The cDNA encoding various Alix domains (Bro1+V, V+PRR, Bro1, and V) were generated by PCR of full-length HA-Alix and constructed with the insertion of an N-terminal HA tag and flanking SalI and BamHI restriction sequences to allow cloning into the expression plasmid pHCMV1 (Genlantis). Forward primers: Bro1+V and Bro1– AGTAGTGTCGACGCGACATTCATCTCGGTGCAGCTGAAAAAG; V+PRR and V – AGTAGTGTCGACTCAGTACAGCAGTCTTTGGCTGCCTATAATCAGAGGAAAG. Reverse primers: Bro1+V and V – AGTAGTGGATCCTCTTTCTGTCTTCCGTGCAAAAACTATATCACTGCATTTGTT; V+PRR – AGTAGTGGATCCCTGCTGTGGATAGTAAGACTGCTGTGGGGG; Bro1–AGTAGTGGATCCCATCTTCTCAAACAGATCAGTAAATTTCTGACTGATGGG.

The Alix^I212D^ and Alix^Y319F^ mutants were generated using QuikChange II XL (Agilent) site-directed mutagenesis. Forward primers: Alix^I212D^ –ACAAGAGATAAAATGAAAGATGCCGACATAGCTAAATTGGCTAATCAGGC; Alix^Y319F^ –TGCAGCAAAGAAGGATAATGACTTCATTTTTCATGATCGAGTTCC. Reverse primers: Alix^I212D^ –GCCTGATTAGCCAATTTAGCTATGTCGGCATCTTTCATTTTATCTCTTGT; Alix^Y319F^ –GGAACTCGATCATGAAAAATGAAGTCATTATCCTTCTTTGCTGCA.

The Flag-MDA5 construct, which expresses human MDA5 with a N-terminal Flag tag from the plasmid pEF-BOS, was kindly provided by Takashi Fujita (Laboratory of Molecular Genetics, Kyoto University) [33]. The HA-Ub, HA-Ub_K48_, and HA-Ub_K63_ constructs, which express human wild-type ubiquitin, ubiquitin in which every Lys is substituted with Arg except Lys-48, and ubiquitin in which every Lys is substituted with Arg except Lys-63, respectively, with a N-terminal HA tag from the plasmid pRK5, were designed in the laboratory of Ted Dawson (Institute for Cell Engineering, Johns Hopkins University) and obtained from Addgene [34]. The Flag-Chmp4b construct, which expresses human Chmp4b containing a N-terminal Flag tag from the plasmid pCMV, was kindly provided by Masatoshi Maki (Laboratory of Molecular and Cellular Regulation, Nagoya University) [35]. Nucleotide sequences were confirmed by using BigDye terminator and a DNA analyzer 3730 (Applied Biosystems).

### DNA Transfection, siRNA Transfection, and Inhibitor Treatment

For transient expression assays, approximately 1,600,000 293 T cells per well of a 6-well plate were transfected with a mixture of 12 µl Lipofectamine 2000 (Invitrogen) per 4 µg of total DNA in 600 µL antibiotic-free OptiMEM. 400 µL 5% FCS DMEM was added at 24 h post-transfection, and the cells were incubated for a total of 48 h at 37°C. For stimulation with poly(I:C) (Sigma), the same number of cells were transfected first with Flag-MDA5 in 1 mL of antibiotic-free OptiMEM, the media was removed at 48 h, and the same cells were then transfected with a mixture of 12 µl Lipofectamine 2000 per 2 µg of poly(I:C) for an additional 6 h at 37°C.

For the knock-down experiments, the same number of cells were transfected with a mixture of 0.1 nmol of siRNA (Alix cat# L-004233-00-0005; Chmp4b cat#L-018075-01-0005; GAPDH cat#L-004253-00-0005; and control non-targeting pool cat#D-001810-10-05 from Dharmacon) and 2.5 µL of DharmaFECT I in 1 mL of 5% FCS antibiotic-free DMEM and incubated for 48 h at 37°C. The cells were split 1∶3 with TrypLE Select, allowed to adhere for 8 h, and transfected with siRNA as above for a second time with siRNA for another 48 h at 37°C in order to increase the efficiency of the knock-down.

Stable A549-derived cell lines were generated in which Alix expression was constitutively knocked-down using shRNA. Cell lines Alix knock-down (kd) #1 and control (ctl) kd #1 were generated using Polybrene infection reagent to transduce commercially available preparations of lentiviral particles (Santa Cruz, cat# sc-60149-V and sc-108080, respectively) into A549 cells. Cell lines Alix kd #2 and ctl kd #2 were generated using lipofectamine to transfect plasmids expressing Alix-specific or control shRNA (Origene Clone ID TRCN0000029396 and TRCN0000029398, respectively) into A549 cells. Successfully transduced/transfected cells were selected with puromycin (1 µg/mL) in 10% FCS DMEM. Individual colonies were then isolated using cloning cylinders and expanded in the presence of puromycin.

To evaluate the effect of various inhibitors of proteolysis on C protein expression, A549 cells were infected with WT HPIV1 and, beginning at 42 h post-infection, treated for 6 h with the proteasome inhibitor MG132 (20 µM), the pan-caspase inhibitor zVAD-FMK (40 µM), or the lysosomal protease inhibitors leupeptin (0.25 mM), E64d (10 µg/mL), pepstatin (10 µg/mL), or bafilomycin (0.5 µM; all from Sigma), and then harvested at 48 h post-infection. 293 T cells transfected with expression constructs were also treated for 6 h with the proteasome inhibitor lactacystin (10 µM; Sigma) beginning at 42 h post-transfection, and the cells were harvested at 48 h post-transfection.

### Immunoprecipitation and Western Blotting

For the preparation of cellular extracts for immunoprecipitation of Western blotting, approximately 1,200,000 cells were suspended in 800 µL of lysis buffer (1% Triton X-100, 50 mM Tris HCl [pH 7.4], 150 mM NaCl, 1 mM EDTA) containing protease inhibitor cocktail (Sigma) for 30 min at 4°C and clarified by centrifugation at 15,000×*g* for 10 min.

For immunoprecipitation, cell lysates were mixed for 16 h at 4°C with anti-Myc or anti-HA antibody conjugated to agarose (Pierce), washed extensively with wash buffer (50 mM Tris HCl [pH 7.4], 150 mM NaCl), and eluted with 0.1 M glycine (pH 2.8).

Cell lysates and immunoprecipitated samples (15 µl) were denatured and reduced in 5 µL of 0.3 M Tris HCl [pH 6.8], 5% SDS, 50% glycerol, and 1 µl of 0.1 M dithiothreitol [DTT]) at 95°C for 5 min. The samples were separated by SDS-PAGE on 4 to 20% Tris-glycine gels (Invitrogen) and were transferred to 0.45-µm-pore polyvinylidene difluoride (PVDF) membranes (Invitrogen). Membranes were blocked overnight with 5% milk powder and 1% bovine serum albumin (BSA) at 4°C. We previously described the preparation of rabbit polyclonal antisera that recognize: (i) the C’, C, Y1, and Y2 proteins; (ii) the N protein; or (iii) the P protein [17]. Membranes were probed with rabbit antiserum at a 1∶1,000 dilution with 5% milk powder and 1% BSA in 0.1% Tween-PBS for 1 h. The antibodies used also included rabbit polyclonal antibodies to Flag epitope tag (F7425; Sigma), hemagglutinin (HA) epitope tag (H6908; Sigma), and Myc epitope tag (C3956; Sigma), goat polyclonal antibodies to Alix (sc-49268; Santa Cruz Biotechnology), and mouse monoclonal antibodies to GAPDH (G9295; Sigma). Horseradish peroxidase-conjugated goat anti-rabbit, donkey anti-goat, and bovine anti-mouse secondary antibodies (Santa Cruz Biotechnology) were used at dilutions of 1∶5000, 1∶5000, and 1∶1000, respectively, for 1 h. Membranes were washed three times for 5 min with 0.1% Tween-PBS after primary and secondary antibody incubations. Enhanced chemiluminescent substrate (Pierce) was used to visualize proteins on BIOMAX MR film (Kodak).

### Immunofluorescence

293 T cells cultured on poly-d-lysine coated coverslips (Becton Dickinson) were fixed 48 h post-infection with 2% paraformaldehyde, permeabilized with 0.1% Triton X-100, and blocked with 5% goat serum, 0.5% BSA, and 0.5% gelatin overnight at 4°C. Mouse monoclonal anti-Alix antibody (H00010015-M01; Abnova) at a 1∶200 dilution, rabbit polyclonal anti-HPIV1 C antibody at a dilution of 1∶350, mouse monoclonal anti-mannose-6-phosphate receptor (M6PR) IgG1 antibody (ab8093; Abcam) at a dilution of 1∶350, and mouse monoclonal anti-early endosome antigen 1 (EEA1) IgG1 antibody (ab70521; Abcam) at a dilution of 1∶500 were added in blocking solution for 1 h. Dylight 594-conjugated goat anti-rabbit, fluorescein isothiocyanate (FITC)-conjugated goat anti-mouse IgG1, and Cy5-conjugated goat anti-mouse IgG2a secondary antibodies (Jackson ImmunoResearch) were added at dilutions of 1∶1000, 1∶200, and 1∶400, respectively, for 45 min. Cells were mounted in ProLong Gold reagent with DAPI (4′,6-diamidino-2-phenylindole; Invitrogen) and visualized on a Leica SP5 confocal microscope.

### Mass Spectrometry

Cell lysates were subjected to SDS-PAGE on duplicate 10% Bis-Tris gels. The first gel was either stained with silver (Invitrogen) to detect total proteins or transferred to PVDF and probed with anti-HA antibody to detect ubiquitinated proteins. The second gel was stained with Coomassie (Invitrogen). Specific bands observed in the first gel were excised from the second Coomassie-stained gel, digested with trypsin, loaded in a LTQ-Orbitrap XL (Thermo Scientific), and analyzed with MASCOT (Matrix Science) and Scaffold (Proteome Software).

### Quantitative PCR

Intracellular RNA was extracted and analyzed by reverse transcription (RT) and quantitative (q) PCR as described previously [10]. GAPDH mRNA levels were determined using a commercially available TaqMan gene expression assay (Applied Biosystems; Hs99999905_m1). HPIV1 P/C mRNA levels were determined by RT with an oligo(dT) primer and performing PCR with the forward primer GACAGAAAGATGGCTGAGAACTCT, reverse primer TCATCAGATAAGGGTGTACTTCTTCGT, and probe CAAGTTGAGGGATTTCC. Experiments were performed in triplicate. Student’s *t* test was used to determine statistical significance.

## Results

### Screening for Cellular Proteins that Interact with the HPIV1 C Proteins

The HPIV1 C proteins (C’, C, Y1, and Y2) are carboxy-coterminal species that are expressed from alternative start codons nested in the same open reading frame of the HPIV1 P/C gene. We designed plasmids to express the C’, C, Y1, and Y2 coding regions, each containing a C-terminal Myc tag (**Fig. 1A**). Additionally, we designed a version of the C’ construct bearing the F170S mutation, and designed versions of the WT and F170S C’ constructs in which an ATG codon was placed immediately before the native GTG start codon. This was done to enhance the expression of the C’ isoform at the expense of the downstream C, Y1, and Y2 isoforms which otherwise are expressed by ribosomes that bypass the native GTG start codon. In addition, the native ACG start codon of Y2 was replaced with an ATG codon to enhance expression.

To identify host proteins that might interact with the HPIV1 C proteins, the C’, C, and Y2 expression constructs were transfected into 293 T cells for 48 h, and the Myc-tagged C proteins were isolated by immunoprecipitation, separated by SDS-PAGE, and visualized by silver staining (**Fig. 1B**). In addition, a second set of 293 T cells were the same C expression constructs transfected along with MDA5 for 48 h and then subsequently transfected the synthetic dsRNA analog poly I:C. This was done because an interaction between the HPIV1 C proteins and host cellular proteins might have depended on changes in signal transduction and cellular gene expression induced during viral infection, and we had previously found that the host cell response to HPIV1 infection relied mainly on MDA5 [14]. Silver staining showed that a species of approximately 105 kDa in size appeared to co-immunoprecipitate with the C’, C, and Y2 proteins, regardless of activation by MDA5 and poly I:C (**Fig. 1B**). The identity of this protein was investigated by in-gel trypsin digestion and mass spectrometry, and the resulting peptide sequences were found to correspond exactly with the human protein Alix (**Fig. 1C**).

In addition, we detected an interaction between Alix and the Y2 protein using a yeast-two-hybrid system (data not shown). We also confirmed that endogenous Alix co-immunoprecipitated with the plasmid-expressed Myc-tagged C’, C, and Y2 proteins regardless of activation by MDA5 and poly I:C (**Fig. 1D**). Alix also co-immunoprecipitated with the Myc-tagged C’ protein containing the F170S mutation (C’^F170S^) but to a much lesser extent (**Fig. 1D**; last two lanes).

**Figure 2:**
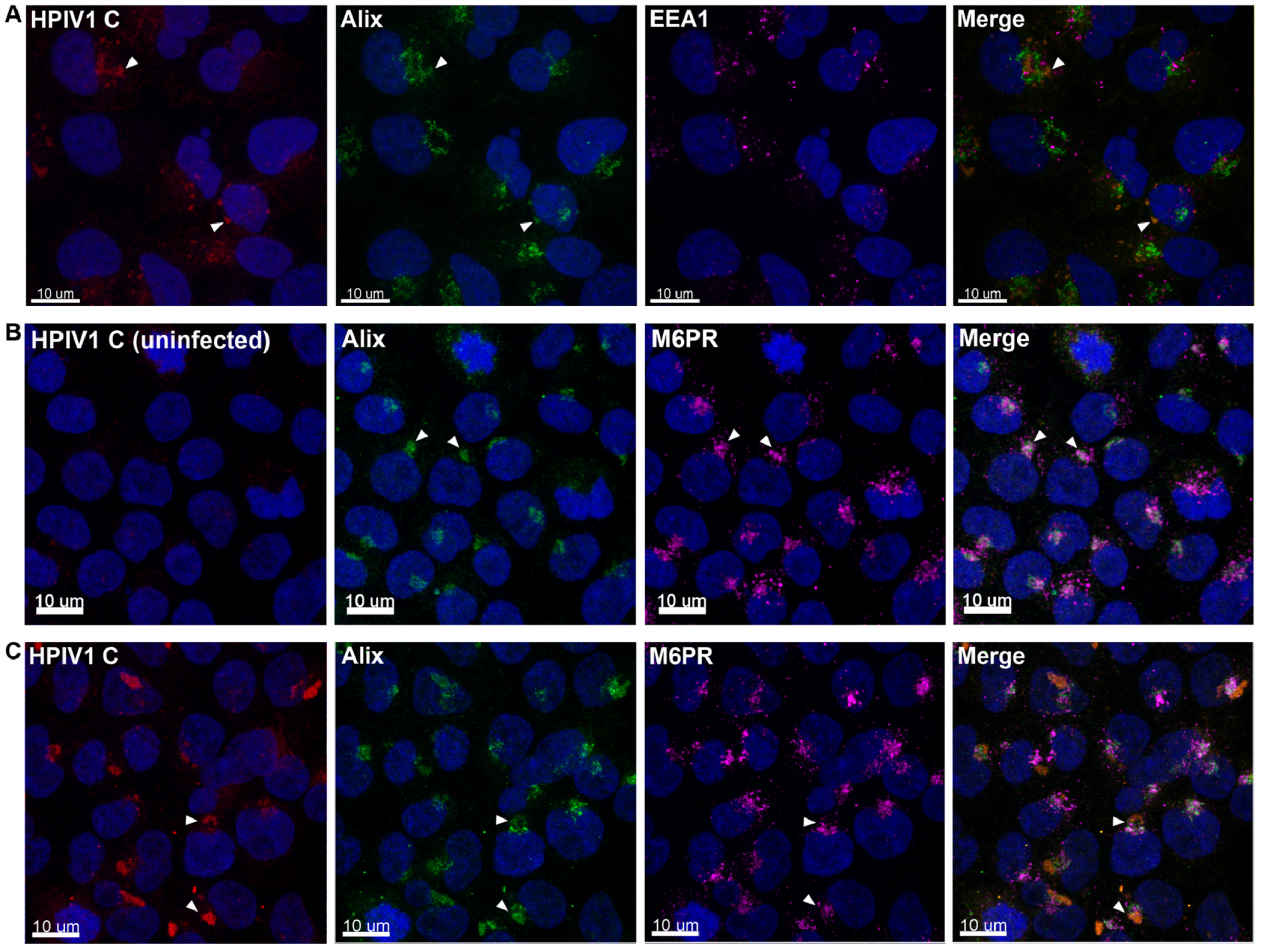
Intracellular co-localization of the C proteins with endogenous Alix and the late endosomal compartment during HPIV1 infection. 293 T cells were mock-infected (**B**) or infected with WT HPIV1 (**A** and **C**) and after 48 h of incubation the cells were fixed, permeabilized, and analyzed by immunofluorescence with the antibodies indicated in each panel. Nuclei were visualized with DAPI staining (blue). **A.** The C proteins (red) and Alix (green), but not the early endosomal marker EEA1 (magenta), co-localized (orange) in HPIV1-infected cells. **B.** Alix (green) and the late endosomal marker M6PR (magenta) co-localized (gray) in un-infected cells. **C.** The C proteins (red), Alix (green), and M6PR (magenta) co-localized (light orange) in HPIV1-infected cells.

Immunofluorescence staining showed that, during HPIV1 infection, the C proteins co-localized with endogenous Alix whereas neither protein co-localized with the early endosomal marker EEA1 (**Fig. 2A** **)**. Also, endogenous Alix co-localized with the late endosomal marker M6PR in uninfected cells (**Fig. 2B** **)**, In cells infected with WT HPIV1, the C proteins co-localized with endogenous Alix and M6PR at the perinuclear region (**Fig. 2C**). In cells infected with HPIV1 containing the C^F170S^ mutation, the C proteins still co-localized with endogenous Alix and M6PR at the perinuclear region (**Fig. S1**).

### The CHMP4b Binding Site in the Bro1 Domain of Alix is Required for Interaction with the HPIV1 C Proteins

**Figure 3:**
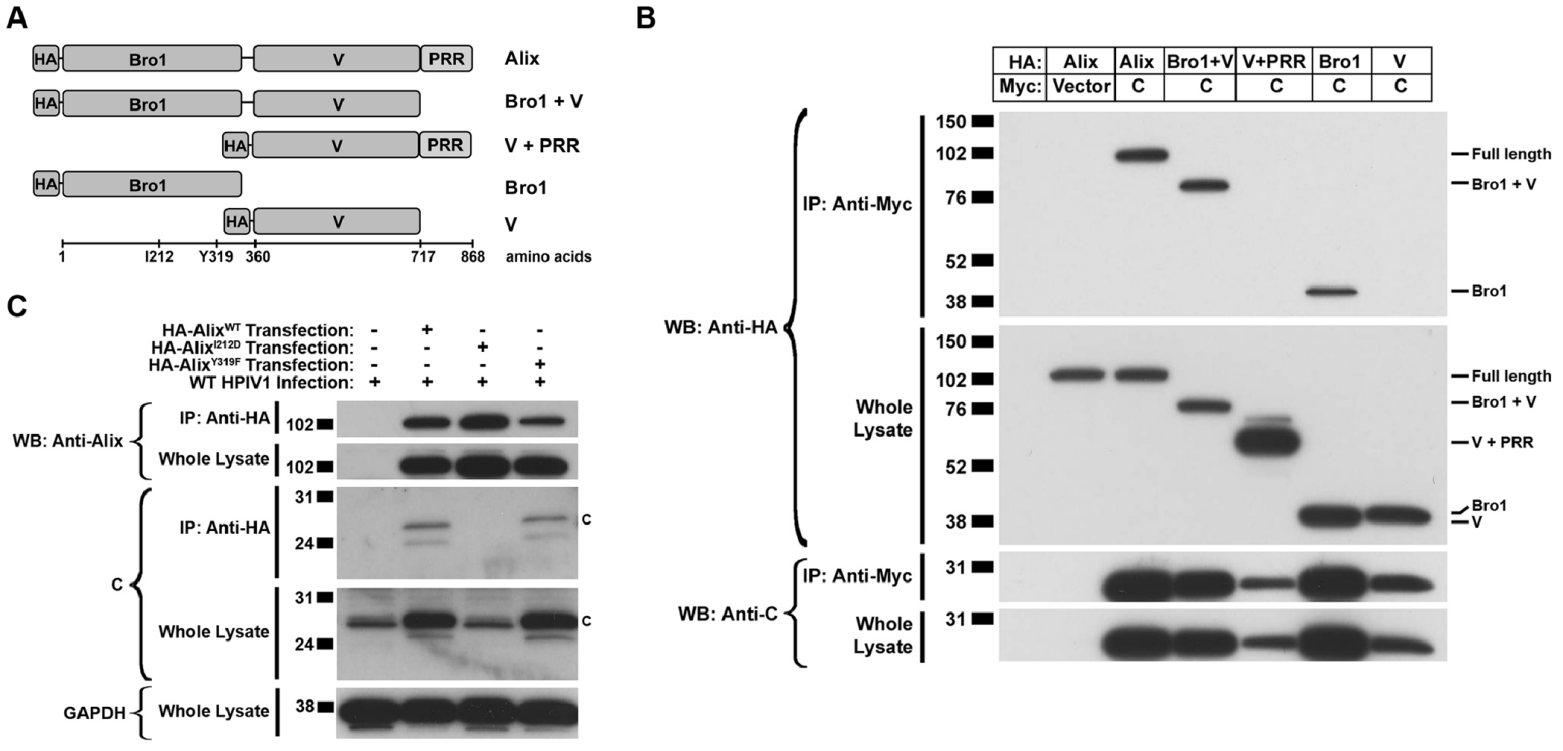
Identification of Alix domains that interact with the C protein. **A.** Schematic diagram of N-terminal HA-tagged Alix domain expression constructs. **B.** Co-immunoprecipitation of the HPIV1 C protein and Alix domains. 293 T cells were co-transfected with plasmids expressing Myc-tagged C protein and the indicated HA-tagged Alix domains. The cells were lysed, subjected to immunoprecipitation using anti-Myc antibodies to isolate the C proteins, and then separated by SDS-PAGE and visualized by Western blotting. Only those Alix constructs containing the Bro1 domain interacted with the HPIV1 C protein. **C.** Effect of the I212D and Y319F mutations in the Bro1 domain on the interaction between Alix and the C protein. 293 T cells were transfected with HA-tagged wild-type Alix or Alix with mutations in the Chmp4 (I212D) or Src SH2 (Y319F) binding sites. 48 h later, the cells were infected with WT HPIV1 and incubated for another 24 h, and cell lysates were prepared and subjected to immunoprecipitation with anti-HA antibodies to isolate Alix. The immunoprecipitated proteins were separated by SDS-PAGE and visualized by Western blotting. This showed that the Chmp4 binding site mutation (I212D), but not the Src binding site mutation (Y319F), abolished the interaction between Alix and the HPIV1 C proteins.

We next attempted to identify site on Alix that binds to the C proteins. Alix is a modular protein of 868 amino acids that consists of 3 major domains: an N-terminal Bro1 domain (residues 1–359) and a C-terminal Proline-rich Region (PRR; residues 717–868) linked by an intervening V-shaped V domain (residues 360–716) (**Fig. 3A**). Expression constructs were designed that encoded the full-length human Alix protein, the Bro1 and V domains together, the V and PRR domains together, the Bro1 domain alone, and the V domain alone, each bearing a N-terminal HA tag (**Fig. 3A**). We found that each of the Alix constructs that contained the Bro1 domain co-immunoprecipitated with the Myc-tagged C protein expressed from a co-transfected plasmid, whereas any Alix construct that lacked the Bro1 domain did not (**Fig. 3B**). The Bro1 domain contains two known binding sites for two cellular proteins that can be abolished by specific point mutations: I212D inactivates the binding site for the CHMP4b protein and Y319F inactivates the binding site for Src kinase. In HPIV1-infected cells, Alix containing the Src kinase binding site mutation Y319F co-immunoprecipitated with the HPIV1 C protein, whereas Alix containing the CHMP4b binding site mutation I212D did not, indicating that an intact Chmp4b binding site in Alix is required for binding the HPIV1 C proteins (**Fig. 3C**).

### The Ability of Alix to Bind C affects the Level of C Protein Accumulation during HPIV1 Infection

**Figure 4:**
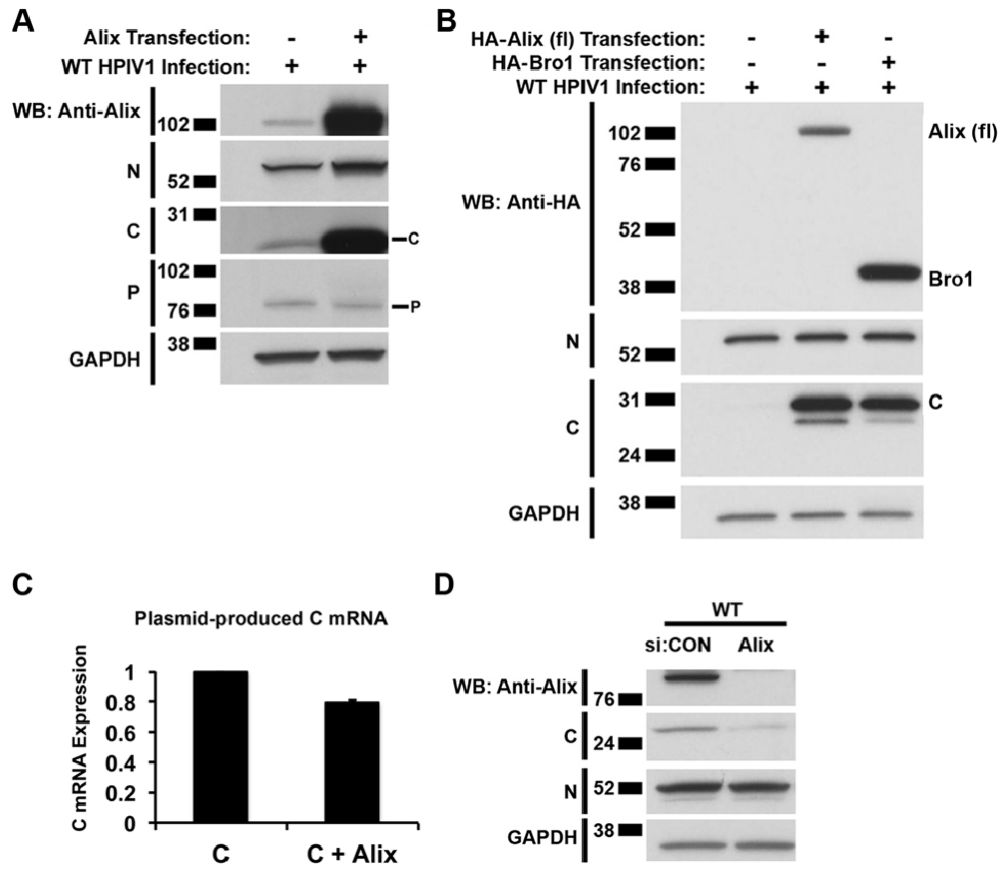
Expression of Alix increases the accumulation of the C protein. **A.** 293 T cells were mock-transfected or transfected with a plasmid expressing the Alix protein. 48 h later, the cells were infected with WT HPIV1, and, after another 24 h, cell lysates were prepared and analyzed by SDS-PAGE and Western blotting with the indicated antibodies. Over-expression of Alix from a transfected plasmid during HPIV1 infection increased the accumulation of the C protein without affecting that of the N and P proteins. Full-length (fl) Alix over-expression in 293 T cells led to substantially increased C protein expression during WT HPIV1 infection. **B.** Using the same experimental protocol as in part A, the Bro1 domain was over-expressed, and it was sufficient to increase C protein expression during HPIV1 infection. **C.** The fl Alix and C expression constructs were co-transfected into 293 T cells, RNA was harvested at 48 h, and plasmid-produced C mRNA levels were measured by RT-qPCR. The increase in the abundance of plasmid-produced HPIV1 C proteins when Alix was over-expressed (**Fig. 3B bottom panel**) was not due to an increase in C mRNA levels. **D.** Knocking down endogenous Alix expression during HPIV1 infection reduced the accumulation of C protein with no effect on the N protein. Cells were transfected with control (CON) or Alix-specific siRNA as described in the Materials and Methods. The cells were then infected with WT HPIV1 and incubated for 24 h. Cell lysates were prepared and analyzed by SDS-PAGE and Western blotting using the indicated antibodies.

In the above experiments, in which different combinations of the three Alix domains were co-expressed with the HPIV1 C proteins, we noticed a substantial increase in the level of intracellular C protein whenever C was co-expressed with any form of Alix bearing the Bro1 domain (**Fig. 3B and C**). This was observed with C protein expressed from transfected plasmid (**Fig. 3B**) and expressed during HPIV1 infection (**Fig. 3C**). Whereas the expression of Alix from transfected plasmid in HPIV1-infected cells caused a dramatic increase in the level of C protein, the levels of N and P proteins were unaffected (**Fig. 4A**). Over-expression of the Bro1 domain alone in HPIV1-infected cells was sufficient for inducing this increase in the level of C protein (**Fig. 4B**). This increase in C protein expression was not due to changes in the accumulation of P/C mRNA, as measured by RT-qPCR (**Fig. 4C**). In addition, when the expression of endogenous Alix in HPIV1-infected cells was reduced by RNA interference, the level of C protein was also reduced whereas the levels of the N and P proteins were unaffected (**Fig. 4D**).

### HPIV1 C Protein Expression is Regulated by Lys-48 Ubiquitination and Turnover by the Proteasome

**Figure 5:**
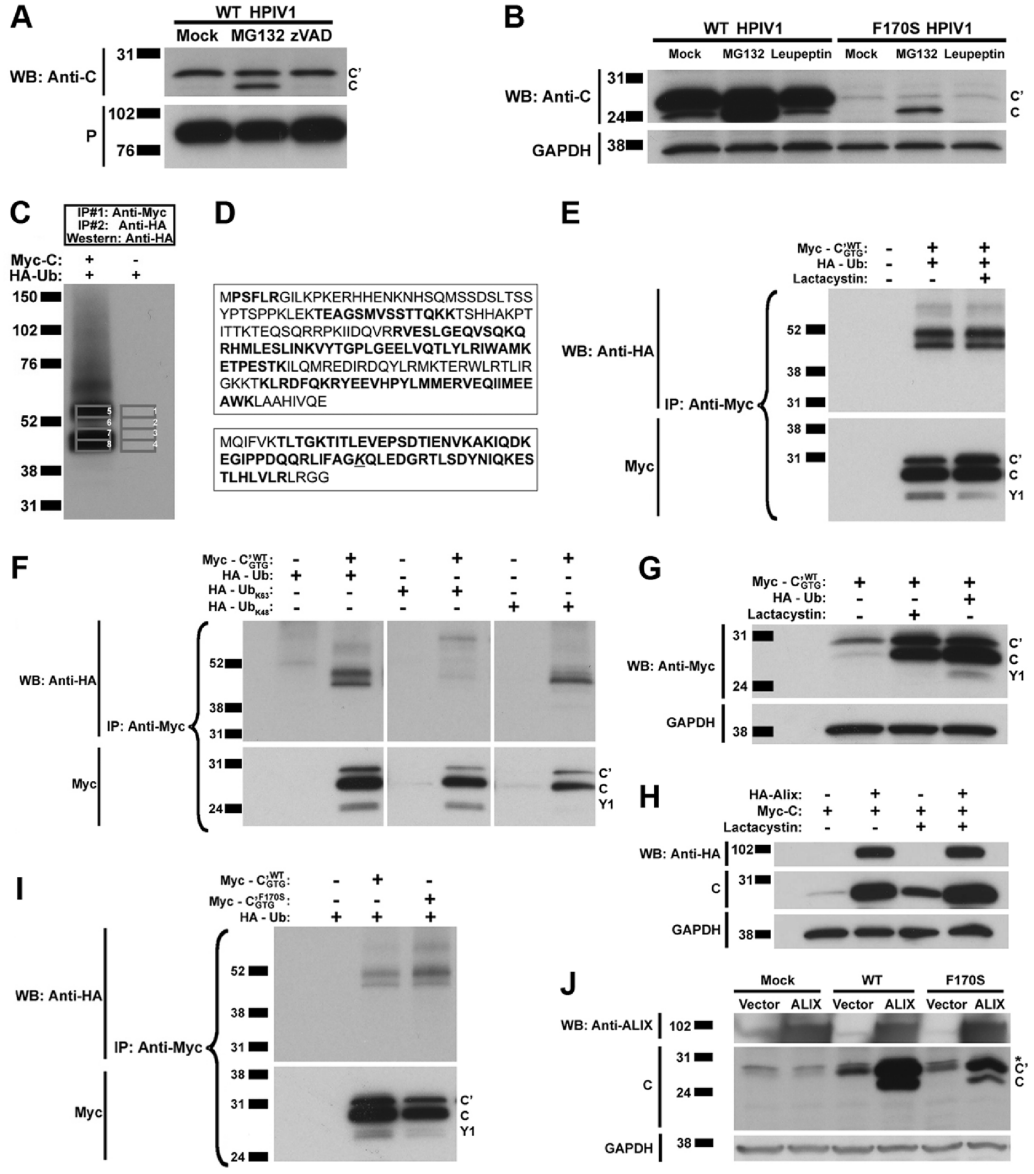
The C protein isoform is subject to proteasome-mediated degradation. **A and B.** Analysis of the effect of various proteolytic inhibitors on C protein accumulation. A549 cells were infected with WT HPIV1 and then treated with the proteasome inhibitor MG132 (**A and B)**, the pan-caspase inhibitor zVAD-FMK (**A**) or the lysosomal protease inhibitor leupeptin (**B)**. Analysis of cell extracts by SDS-PAGE and Western blotting showed that only the proteasome inhibitor MG132 was associated with increased accumulation of the C isoform, whereas the C’ isoform was unaffected. **C and D.** Evidence for ubiquitination of the HPIV1 C protein. **C.** 293 T cells were transfected with plasmids expressing Myc-tagged C protein and HA-tagged ubiquitin (Ub). Two serial immunoprecipitations were performed, the first using anti-Myc antibodies to isolate the C protein, and the second using immobilized anti-HA antibodies to isolate ubiquitinated proteins. The proteins were separated by SDS-PAGE and analyzed by Western blotting to visualize the ubiquitinated proteins. Bands 1–8 were excised from a duplicate gel that had been run in parallel and stained with Coomassie blue, and the gel slices were analyzed using mass spectrometry. Bands 1–4 served as a negative control. **D.** Amino acid sequences of the C protein (top) and ubiquitin (bottom) showing peptide sequences identified by mass spectrometry (bold). In addition, the Lys-48 residue of ubiquitin (underlined), but not Lys-63, was found to have evidence of a Gly-Gly modification corresponding to the presence of a poly-ubiquitin linkage. A Gly-Gly modification (underlined) corresponding to a poly-ubiquitin linkage was found at Lys-48 and not at Lys-63. **E.** Confirmation that the C protein is ubiquitinated. 293 T cells were transfected with Myc-tagged C’^WT^_GTG_ and HA-tagged ubiquitin expression constructs and incubated in the presence or absence of the proteasome inhibitor lactacystin. The Myc-tagged C proteins were isolated by immunoprecipitation using anti-Myc antibodies, separated by SDS-PAGE, and then analyzed by Western blotting. **F.** Confirmation that the poly-ubiquitin linkage involves Lys-48. Plasmids expressing HA-tagged ubiquitin with every lysine mutated into an arginine except for Lys-63 (Ub_K63_) or Lys-48 (Ub_K48_) were transfected into 293 T cells together with plasmid expressing Myc-tagged C’^WT^_GTG_. Cell lysates were subjected to immunoprecipitation with anti-Myc antibodies to isolate the C protein and analyzed by SDS-PAGE and Western blotting to detect the presence of poly-ubiquitin in the high molecular weight bands. **G.** The accumulation of C protein isoforms was increased by the over-expression of ubiquitin to a level comparable to that seen after treatment with a proteasome inhibitor. 293 T cells were transfected with HA-tagged ubiquitin and Myc-tagged C and subsequently treated with the proteasome inhibitor lactacystin. C protein expression was examined by Western blotting. **H.** The accumulation of C protein was also increased by the over-expression of Alix to a level comparable to that seen after treatment with a proteasome inhibitor. 293 T cells were transfected with HA-tagged Alix and Myc-tagged C and subsequently treated with the proteasome inhibitor lactacystin. C protein expression was examined by Western blotting as in part G. **I.** The F170S mutation-containing C proteins also undergo ubiquitination. 293 T cells were transfected with plasmid expressing HA-tagged ubiquitin with plasmids expressing Myc-tagged C’^WT^_GTG_ or C’^F170S^_GTG_. As in panel **F**, cell lysates were subjected to immunoprecipitation with anti-Myc antibodies to isolate the C protein and analyzed by SDS-PAGE and Western blotting to detect the presence of poly-ubiquitin in the high molecular weight bands. **J.** Alix over-expression increases the abundance of F170S mutation-containing C proteins but to a lesser extent compared to the WT C proteins. 293 T cells were transfected with plasmid expressing Alix and then infected with WT of F170S HPIV1. C protein expression was then examined by Western blotting. The * represents a non-specific band.

Given the observation that the intracellular accumulation of C was increased by the co-expression of Alix, we decided to investigate C protein stability. During an infection of 293 T cells with HPIV1, the C protein was the predominant C isoform and little or no C’ was observed, as shown in previous experiments (e.g. **Fig. 3C** and data not shown). In contrast, during an infection of A549 cells with HPIV1, the C’ protein was the predominant isoform and little or no C protein was detected (**Fig. 5A**). However, treatment of HPIV1-infected A549 cells with a proteasome inhibitor, like MG132, substantially increased the level of C protein without having any effect on C’ or P protein levels (**Fig. 5A**). In contrast, the pan-caspase inhibitor zVAD-FMK (**Fig. 5A**), the lysosomal protease inhibitors leupeptin (**Fig. 5B**), E64d, pepstatin, and bafilomycin (not shown) did not have an effect on C protein levels. Together, this indicated that the accumulation of C protein was affected by proteasome-mediated degradation.

Since ubiquitination, specifically Lys-48 linked polyubiquitination, targets a protein for proteasome-mediated degradation, we examined the ubiquitination state of the HPIV1 C proteins. 293 T cells were co-transfected with plasmids expressing Myc-tagged C protein and HA-tagged ubiquitin. Immunoprecipitation was performed with anti-Myc antibodies to isolate C proteins, and a second round of immunoprecipitation was performed with anti-HA antibodies to isolate proteins from the first immunoprecipitation that had been ubiquitinated with plasmid-expressed ubiquitin. The isolated proteins were subjected to SDS-PAGE and analyzed by Western blotting with anti-HA antibodies to detect ubiquitinated species. This detected two predominant bands in the size range of 45–60 kDa, as well as a less abundant heterodisperse smear of larger species. The difference in apparent molecular weights between the two major ubiquitinated species and the ∼31 kDa unmodified C’ protein suggested that the C proteins were conjugated to 2–3 ubiquitin moieties. The bands between 45–60 kDa were excised, digested with trypsin, and analyzed by mass spectrometry (**Fig. 5C**). The peptide sequences corresponded to ubiquitin and the HPIV1 C proteins (**Fig. 5D**). Furthermore, a Gly-Gly modification was found on Lys-48 in the sequence for ubiquitin: this was evidence of linkage to the C-terminus of a second ubiquitin moiety and was consistent with polyubiquitination involving Lys-48.

When the C proteins were immunoprecipitated from cells co-expressing the Myc-tagged C proteins with HA-tagged ubiquitin from transfected plasmids, similar high molecular weight bands containing HA-tagged polyubiquitin also were detected (**Fig. 5E**). To further investigate whether these polyubiquitin linkages involved Lys-48 or Lys-63, 293 T cells were co-transfected with plasmids expressing Myc-tagged **C’^WT^_GTG_** and HA-tagged ubiquitin mutants in which every Lys residue had been substituted with Arg except for Lys-48 (HA-Ub_K48_) or Lys-63 (HA-Ub_K63_) (**Fig. 5F**). The polyubiquitinated C proteins were observed only when Lys-48 was present (wild-type HA-Ub or HA-Ub_K48_), confirming the presence of Lys-48 linkages in C protein polyubiquitination.

Incidentally, in these experiments, we noticed that over-expression of ubiquitin resulted in a substantial increase in the accumulation of the C proteins (**Fig. 5G**). It may be that the over-expression of ubiquitin and the resulting increase in the size of the ubiquitin pool led to an accumulation of ubiquitinated proteins, including autoubiquitinated ubiquitin ligases, that overwhelmed the cellular capacity for proteasome-mediated degradation [36]. Consistent with this suggestion, the further inclusion of a proteasome inhibitor did not confer a further increase in the abundance of C proteins (**Fig. 5G**), indicating that a lack of proteasome-mediated degradation of the C proteins in the presence of ubiquitin over-expression. The over-expression of the C-interacting protein Alix, as described above (**Fig. 3B**), also increased the accumulation of C protein to comparable levels (**Fig. 5H**).

We found that the level of mutant C^F170S^ proteins was also modulated by proteasome-mediated degradation, ubiquitination, and Alix over-expression, albeit to a lesser extent compared to the WT C proteins. During infection with HPIV1, less of the C^F170S^ proteins were detected compared to the WT C proteins (**Fig. 5B**). Treatment of HPIV1-infected A549 cells with the proteasome inhibitor MG132 also increased the level of C^F170S^ protein without having any effect on C’ protein levels (**Fig. 5B**). When the C^F170S^ and WT C proteins were immunoprecipitated from cells co-expressing the Myc-tagged C proteins with HA-tagged ubiquitin, similar high molecular weight bands containing HA-tagged polyubiquitin were also detected (**Fig. 5I**). Since the interaction between C^F170S^ and Alix is significantly weaker than the interaction between WT C and Alix, we decided to test whether the effect of Alix expression on C^F170S^ protein levels was blunted. Although Alix over-expression increased the abundance of both the WT and F170S mutation-containing C proteins, the level of WT C proteins was increased to a much greater extent (**Fig. 5J**).

### Changes in the Level of Alix Expression do not Affect the Efficiency of HPIV1 Replication or Release

**Figure 6:**
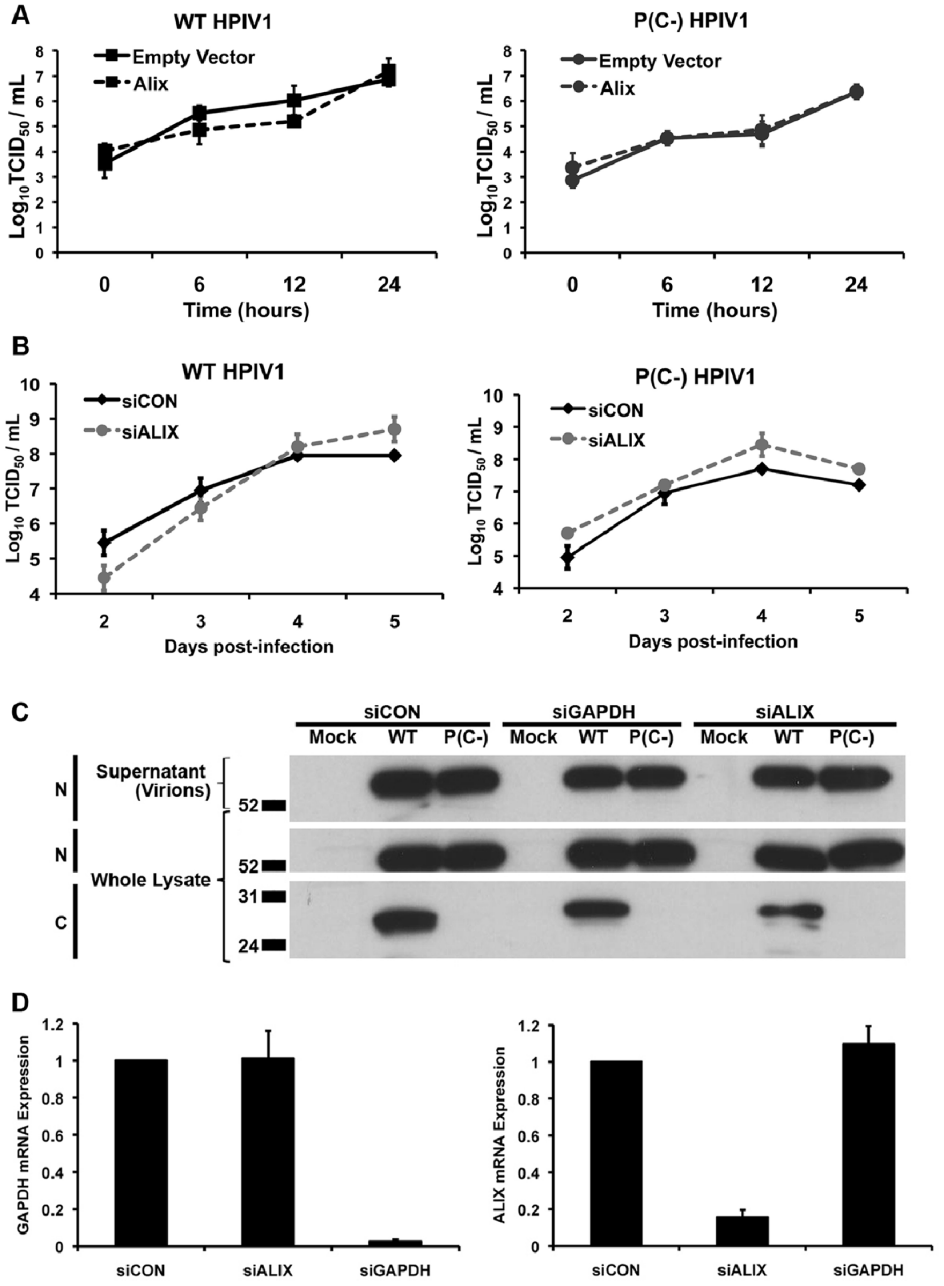
Changes in the expression of Alix do not alter HPIV1 replication or release. **A.** Over-expression of Alix did not affect HPIV1 replication. 293 T cells were transfected with plasmid expressing Alix and then infected with WT or P(C-) HPIV1 at a MOI of 5 (the same MOI that was used in previous experiments). 0.5 mL was aliquoted from the overlying tissue culture medium and replaced with 0.5 mL fresh medium at the indicated time points. The aliquots were assayed for infectious virus by limiting dilution. **B.** siRNA-mediated knock-down of Alix expression did not affect HPIV1 replication. 293 T cells were transfected twice with control non-targeting (siCON) or Alix-specific (siALIX) siRNA and infected with a low MOI (0.01) of WT or P(C-) HPIV1. HPIV1 replication was assayed as in part A. **C.** Analysis of viral proteins released into the overlying tissue culture medium or present intracellularly during Alix knock-down. On day 5 of the experiment described in part B (with the addition of 293 T cells transfected with siGAPDH as an additional control), total protein from the tissue culture supernatant and from the cell lysate was collected and analyzed by SDS-PAGE and Western blotting. This confirmed the presence of N protein in virions released into the overlying culture medium and in cell lysates for both viruses, the absence of intracellular C protein for the P(C-) virus, and the reduced accumulation of C protein from WT HPIV1 during Alix knock-down. **D.** Confirmation of siRNA-mediated knock-down of Alix expression. From the experiment in part C, the knock-down of Alix and GAPDH expression was confirmed by measuring intracellular mRNA by RT-qPCR.

We next investigated whether Alix played a significant role in the growth and spread of HPIV1. To evaluate the effects of the over-expression of Alix, 293 T cells were transfected with plasmid that expressed Alix and then infected with WT or P(C-) HPIV1 (**Fig. 6A**). The release of infectious virus into the medium overlay was sampled over a 24 h period. This showed that there was essentially no difference in the efficiency or kinetics of WT or P(C-) HPIV1 replication when Alix was over-expressed (**Fig. 6A**).

In addition, the expression of endogenous Alix was knocked-down by RNA interference during HPIV1 infection of 293 T cells (**Fig. 6B**). In this experiment, the HPIV1 infection was done at a lower MOI (0.01 compared to 5.0 used in other experiments) in order to monitor multi-cycle infection, which should provide increased sensitivity for detecting changes to in viral replication or release. However, no difference was observed in the efficiency or kinetics of replication of either WT of P(C-) HPIV1 in response to the reduced expression of Alix **(** **Fig. 6B** **)**. Knocking down Alix also did not have a detectable effect on the amount of viral N protein released from cells into the supernatant (**Fig. 6C**), in concordance with the lack of an effect on the production of infectious particles. RT-qPCR analysis confirmed that the accumulation of Alix mRNA was efficiently reduced by the Alix-specific siRNA (**Fig. 6D**).

We also performed experiments in which endogenous Alix expression was further reduced. We used selectable lentiviral vectors and plasmids expressing Alix-specific shRNA to generate two independent A549-derived cell lines in which Alix expression was permanently and homogeneously reduced. We did not find any difference in the efficiency or kinetics of HPIV1 replication under these conditions (**Fig. S2A and D**). This was also true for human parainfluenza virus type 2 (HPIV2) and SeV, which were examined in parallel (**Fig. S2B and C**).

### Changes in the Level of Chmp4 Expression Affect HPIV1 Replication and Release

**Figure 7:**
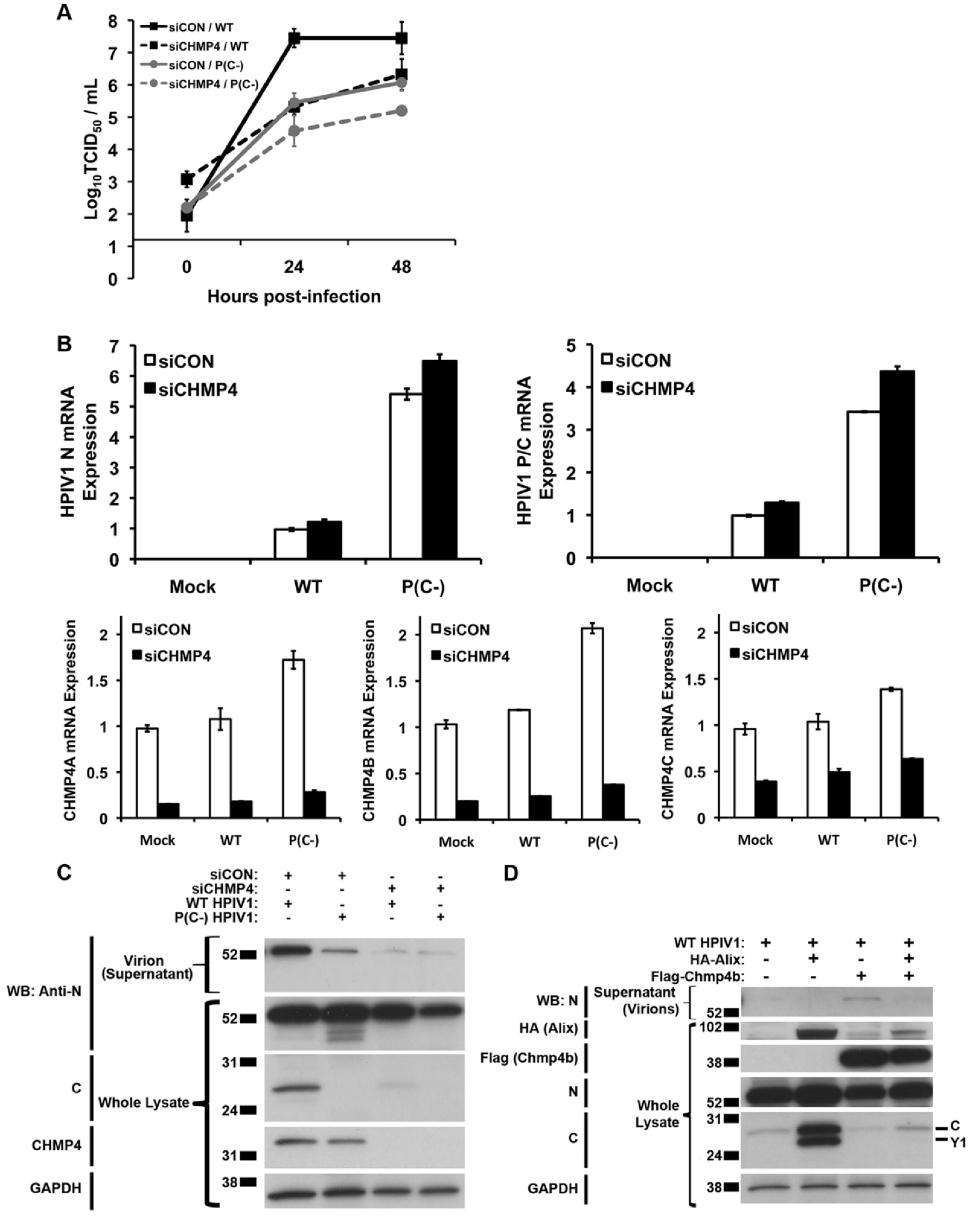
Changes in the expression of Chmp4 alter WT HPIV1 replication and release. **A.** Effect of Chmp4 knock-down on WT HPIV1 replication. 293 T cells were transfected twice with a mixture of siRNAs specific to the Chmp4 isoforms a, b, and c (collectively called siCHMP4) or a control siRNA (siCON) and were infected with WT or P(C-) HPIV1 at a MOI of 5. The release of infectious virus was then quantified by a limiting dilution assay. **B.** Effect of Chmp4 knock-down on the accumulation of intracellular viral and Chmp4 mRNAs. 293 T cells were transfected with siRNA and infected with WT or P(C-) HPIV1 as described in part A. Total RNA was extracted from WT and P(C-) HPIV1-infected 293 T cells after Chmp4 knock-down and reverse transcribed using oligo-dT. Viral N and P/C mRNA and Chmp4a, Chmp4b, and Chmp4c levels were measured by quantitative PCR. **C.** The whole cell lysate and the supernatant were collected from WT and P(C-) HPIV1 infected 293 T cells after Chmp4 knock-down at 48 hours post-infection (p.i.) and Western blotted for the HPIV1 N and C proteins and Chmp4b. **D.** Similarly, the whole cell lysate and the supernatant were collected from WT HPIV1-infected 293 T cells after Alix and/or Chmp4 over-expression at 12 hours post-infection (p.i.) and Western blotted for the HPIV1 N and C proteins and Chmp4b and Alix. Note, during an infection of 293 T cells instead of A549 cells, the C protein, rather than the C’ protein, was the main C isoform.

Since changes in the expression of Alix did not appear to have any discernable effect on HPIV1 replication, we decided to target Chmp4, which is a core component of ESCRT III and binds to the same region of Alix as the HPIV1 C proteins. There are three isoforms of Chmp4 (a, b, and c) that each bind to the Bro1 domain of Alix, with Chmp4b being the principal binding partner of Alix [37]. 293 T cells were transfected twice with a mixture of three pools of siRNAs, each specific to Chmp4 isoforms a, b, and c or with a control siRNA. The cells were infected with WT or P(C-) HPIV1, and the release of infectious virus was monitored. In the control cells, the replication of the P(C-) mutant was delayed and reduced approximately 100-fold compared to WT HPIV1, which is characteristic of this mutant. Interestingly, in the presence of Chmp4 knock-down, the replication of WT HPIV1 was delayed and reduced to the same level observed for the P(C-) HPIV1 mutant in control cells (Fig. 7A). Replication of the P(C-) mutant was reduced further in the presence of Chmp4 knock-down, but the effect was relatively small, ranging from 0.5 to 1 log_10_ (**Fig. 7A**). Analysis of the intracellular levels of N and P/C mRNA by RT-qPCR showed that Chmp4 knock-down had little effect on N and P/C mRNA accumulation, indicating that viral RNA replication and transcription had not been affected (**Fig. 7B**). Notably, the level of N and P/C mRNA expressed by the P(C-) mutant was substantially higher than that of WT HPIV1 irrespective of Chmp4 knock-down; this difference between the two viruses was described previously and shown to be due to increased viral RNA synthesis in the absence of the C protein [14]. The levels of Chmp4a, b, and c mRNA were confirmed by RT-qPCR, and they were substantially reduced by treatment with siRNA (**Fig. 7B**). We also measured the amounts of N protein released in the supernatant and found that these were lower in Chmp4 knock-down cells infected with WT and P(C-) hPIV1 compared to control knock-down cells (**Fig. 7C**), which is in accordance with the reduction in the release of infectious virus already noted (**Fig. 7A**).

We also investigated the effect of over-expressing Chmp4b. 293 T cells were transfected with plasmids expressing Chmp4b and/or Alix, and the cells were infected with HPIV1. At 48 h post-infection, clarified medium supernatants and whole cell lysates were prepared and analyzed by SDS-PAGE and Western blotting (**Fig. 7D**). With the over-expression of Chmp4b alone, an increase in N protein released in the supernatant was observed (**Fig. 7D**). As already described, the over-expression of Alix alone resulted in a large increase in the accumulation of C protein due to the ability of Alix to spare the C protein from proteasome-mediated degradation. In contrast, over-expression of Chmp4b alone in HPIV1-infected cells had no effect on the level of C protein (**Fig. 7D**). Interestingly, co-expression of Chmp4b together with Alix abrogated the increase in C protein accumulation normally conferred by Alix. This suggested that there is a preferential interaction between Alix and Chmp4b rather than Alix and C.

### The HPIV1 C Proteins and Chmp4b Compete with Each Other to Bind Alix

**Figure 8:**
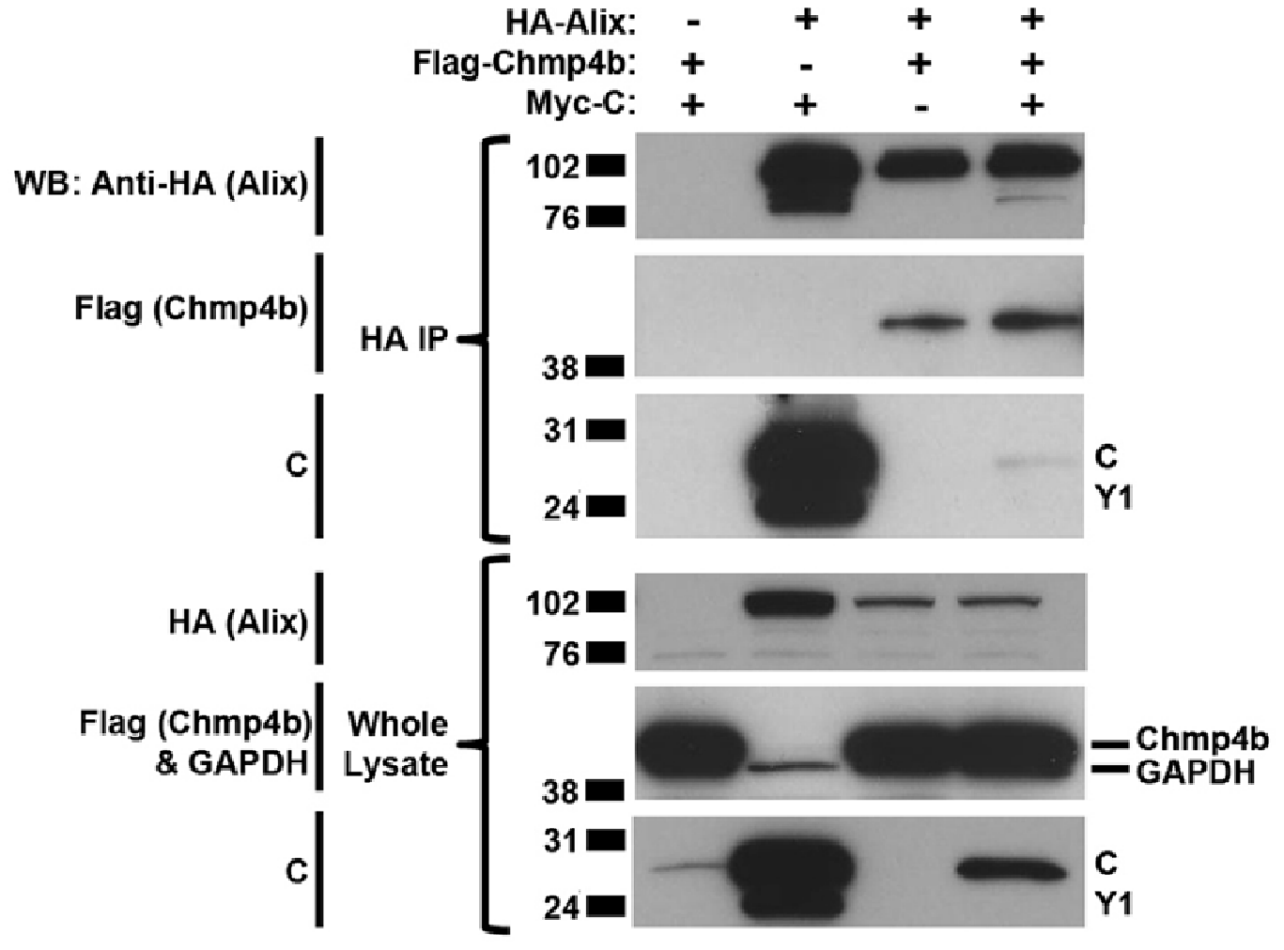
The HPIV1 C proteins and Chmp4b compete with each other to bind Alix. 293 T cells were co-transfected with various combinations of plasmids (as indicated) expressing Myc-tagged C, Flag-tagged Chmp4b, and HA-tagged Alix. 48 h post-transfection, cell lysates were prepared and subjected to immunoprecipitation with anti-HA antibodies to isolate HA-Alix followed by SDS-PAGE and Western blotting.

We performed co-immunoprecipitation experiments to investigate the possible competition between the HPIV1 C and Chmp4 proteins for binding Alix. 293 T cells were co-transfected with various combinations of plasmids expressing Myc-tagged C, Flag-tagged Chmp4b, and HA-tagged Alix. Cell lysates were prepared 48 h later and subjected to immunoprecipitation with anti-HA antibodies to isolate Alix followed by SDS-PAGE and Western blotting. This confirmed that the HPIV1 C and Chmp4b proteins individually interacted with Alix, and that Alix greatly increased the accumulation of the C protein (**Fig. 8**). However, when the three proteins were over-expressed together, binding was observed between Chmp4b and Alix but not between Alix and the C protein. Also, the increase in the accumulation of the C protein normally conferred by Alix was mostly blocked (**Fig. 8**).

## Discussion

The HPIV1 C proteins have previously been shown to down-regulate viral RNA synthesis and thereby prevent activation of MDA5 and PKR [14], to sequester STAT1 and thereby inhibit IFN-mediated signaling [17], to affect the expression of hundreds of cellular genes [10], and to suppress apoptosis in an IFN-independent manner [11]. In the present study, we found that the HPIV1 C proteins also interact with the host cell ESCRT system. In particular, the three C protein isoforms that were evaluated, C’, C, and Y2, were found to bind to the cellular protein Alix and, moreover, to compete with the ESCRT-III component Chmp4b for binding Alix. These interactions had a major impact on the stability of intracellular C proteins. Interestingly, Chmp4b, but not Alix alone, was important for efficient production and release of HPIV1 particles.

The cellular ESCRT system sorts cargo and promotes the invagination of endosomal membranes into the lumen to form MVBs, is involved in membrane remodeling during cytokinesis, and can be hijacked by a number of enveloped viruses to promote virion morphogenesis. The Alix and Chmp4b proteins described in the present study are part of the cellular ESCRT system, and the binding between Alix and Chmp4b is highly conserved in eukaryotic cells, including yeast. Alix can serve as an adaptor that recruits both viral and host proteins to specific biological membranes. For example, by binding either directly to Gag or the midbody protein CEP55, Alix is able to recruit Chmp4 to sites of retroviral budding or cellular abscission [18], [19]. Conversely, Chmp4 is able to recruit Alix to endosomal membranes to facilitate MVB formation, and mutating a single residue I212D in the Bro1 domain of Alix abrogates the interaction with Chmp4b [27], [38].

The use of the ESCRT system in virus budding has been studied most extensively for retroviruses, including HIV1. These retroviruses utilize so-called late (L) domains containing a YP(X_n_)L motif present in their Gag proteins to bind to the V domain of Alix. A number of other enveloped viruses have comparable late domain sequences in the M protein. In contrast, the HPIV1 C proteins have no such motif. The introduction of the F170S mutation into the C proteins, which is present in a live attenuated vaccine currently in phase I clinical trials, was found to weaken the interaction with Alix (**Fig. 1D**), suggesting that this residue is important for the integrity of the binding site. However, this mutation in C does not lie in any known functional region or sequence motif. The interaction between Alix and the HPIV1 C proteins was found to occur within the Bro1 domain of Alix (**Fig. 3**) and, thus, differs from the situation for retrovirus Gag proteins, which bind to the V domain of Alix. Furthermore, the interaction between the HPIV1 C proteins and Alix was found to rely on the I212 residue located within an exposed hydrophobic patch on the concave surface of the Bro1 domain. This same site is involved in the interaction between Alix and Chmp4b. In contrast, the site in the Bro1 domain that mediates an interaction with Src kinase and involves residue Y319 was inconsequential for interaction with the HPIV1 C proteins.

Immunofluorescence of HPIV1-infected cells showed that both the WT and F170S mutation-containing C proteins were mainly localized near late endosomes and co-localized with Alix (**Fig. 2**). Interestingly, we had previously shown that, in HPIV1-infected cells, the C proteins sequester the STAT1 transcription factor in aggregates near the late endosomes [17] that appear to be the same as that described in the present study. Thus, the association between the C proteins and Alix and the localization of C near the late endosomes may serve more than one purpose. Specifically, Alix may allow the C proteins to enter the ESCRT system in order to promote budding, as described in the present study, as well as to block IFN signaling. Indeed, C proteins containing the F170S mutation can only weakly bind Alix (**Fig. 1D**), and, although F170S HPIV1 replicates and buds as effectively as WT HPIV1, F170S HPIV1 fails to block interferon signaling [10], [17]. We have similarly shown that the F170S mutation substantially weakens the interaction between the C proteins even though the C proteins still co-localize with STAT1 at the perinuclear region. This is analogous to the interaction between the C proteins and Alix. However, due to the weakened interaction, the C^F170S^ proteins, unlike the C^WT^ proteins are unable to prevent STAT1 nuclear translocation or block interferon signaling [10], [17]. The observation that the C proteins of HPIV1 accumulate mainly in the vicinity of the late endosomes in HPIV1-infected cells is somewhat different than observations with the related murine virus SeV. A number of groups have reported localization of the SeV C proteins in various compartments of the cell, either localizing in the cytoplasm during an active infection or concentrated along the plasma membrane during over-expression of the C proteins [24], [39]. The apparent difference between the C proteins of HPIV1 and SeV may represent an authentic difference, but it also is possible that the use of plasmid-based over-expression in some of the SeV studies resulted in non-authentic localization. Here, we note that our localization studies involved authentic HPIV1 infection in human cells and also in non-human primate cells [17].

In the process of studying the interaction between Alix and the C proteins, we noted that Alix substantially increased the intracellular accumulation of the HPIV1 C proteins during HPIV1 infection or in response to transfected plasmid. Specifically, the accumulation of C protein was substantially increased in response to over-expression of Alix from transfected plasmid and, conversely, C protein accumulation was reduced when the endogenous level of Alix was knocked down by RNA interference (**Fig. 4**). In contrast, changing the level of expression of Alix had no effect on the accumulation of the N and P proteins in HPIV1-infected cells, and, thus, this effect was specific to C. The increased accumulation of C observed in the presence of Alix over-expression was not due to a change in the accumulation of C mRNA. Thus, the effect appeared to be at the level of protein stability. The ability of Alix to increase the accumulation of HPIV1 C proteins mapped to the Bro1 domain of Alix and depended on the intact binding site involving residue I212. Furthermore, the ability of Alix to stabilize the C proteins was largely arrogated by over-expression of Chmp4b, which was shown to compete with C for binding Alix. Thus, stabilization of the C proteins by co-expression of Alix appeared to require binding to the Bro1 domain.

Ubiquitination and proteasome-mediated degradation were found to be involved in determining the stability of the HPIV1 C proteins. Ubiquitination is an important mechanism by which the host cell tags proteins for degradation by either the proteasome or the lysosome, or for signal transduction, or for sorting cargo into endosomes. Ubiquitin is conjugated via its C-terminal glycine to a lysine residue of a target protein by ubiquitin ligases, and ubiquitin itself has multiple lysine residues that can be targeted for linkage with free ubiquitin to form long polyubiquitin chains. Mono- and polyubiquitination can confer various fates and functions to the tagged protein. Lysine-48 linkages are well-known for targeting proteins for degradation via the proteasome, whereas Lysine-63 linkages are involved in endocytosis, in targeting proteins for degradation via the lysosome, and also in regulating signal transduction through the NF-**κ**B pathway [40], [41]. We found that the accumulation of the C protein in HPIV1-infected cells was substantially increased when the cells were treated with any of several proteasome inhibitors. In addition, co-immunoprecipitation followed by mass spectrometry or Western blotting showed that the C proteins were ubiquitinated and also provided evidence of polyubiquitination involving Lysine-48 linkages (**Fig. 5**). The exact lysine residue(s) on the C proteins that is ubiquitinated remains to be determined. It is interesting to note that C protein expression rather than C’ protein expression is most affected by ubiquitination and proteasome-mediated degradation (**Fig. 5A**). The reasons behind this observation are unclear. The C’ isoform has 15 additional N-terminal amino acids compared to the C isoform that might somehow protect the rest of the protein from ubiquitination and degradation. It is clear, however, that the C proteins are ubiquitinated and degraded by the proteasome, and the mechanism by which Alix stabilizes the C protein is dependent on the ability of the Bro1 domain to bind C and protect it from degradation. It is tempting to speculate that the activity of the host cell in targeting C proteins for degradation may be an effort to “clear” the cytoplasm of a viral protein that otherwise inhibits the host innate immune response. Conversely, the ability of the C protein to bind Alix may be an activity that has been selected for because it spares C from degradation.

The C^F170S^ proteins, like the wild-type C proteins, are also subject to proteasome-mediated degradation (Fig. 5B) and ubiquitination (Fig. 5I). Due to the relatively weaker interaction between C^F170S^ and Alix, in contrast to the interaction between WT C and Alix, we expected that the C^F170S^ proteins would be less stable and more susceptible to ubiquitination and proteasome-mediated degradation. Indeed during infection with HPIV1, less C^F170S^ protein was detected compared to WT C protein (Fig. 5B). Although Alix over-expression appeared to protect both the wild-type C and mutant C^F170S^ proteins from degradation and thereby increase their abundance, the level of WT C proteins was increased to a greater extent (Fig. 5J).

Ubiquitination also plays a key role in the sorting of cargo in ESCRT complexes during MVB biogenesis. The ESCRT-0 complex contains several ubiquitin-binding motifs that are capable of clustering ubiquitinated cargo at membranes that subsequently invaginate with the help of ESCRT-II and ESCRT-III into numerous cargo containing buds [38], [42]. ESCRT-I and ESCRT-II form a scaffold at the neck of these buds where ESCRT-III can then assemble. The ESCRT-III complex recruits Bro1 and then Snf7 in yeast (Alix and Chmp4, respectively, in humans), providing a critical scission factor in the final step to forming cargo-containing intraluminal vesicles. Western blot analysis and mass spectrometry suggested that the C proteins were mainly conjugated to short chains consisting of only 2–3 ubiquitin moieties (**Fig. 5**). Although it remains unclear whether ubiquitination serves as a sorting signal for the C proteins, it is conceivable that ubiquitination of the HPIV1 C proteins allows them to take advantage of the host ESCRT machinery, to aggregate near late endosomes, and to possibly initiate budding through the MVB. In yeast, Bro1 interacts with an ubiquitin thiolesterase Doa4 that facilitates the deubiquitination of cargo just before membrane scission releases cargo into the endosomal lumen, and this mechanism may explain why the interaction between Alix_Bro1_ and the HPIV1 C proteins protect the C proteins from degradation by the proteasome [43].

Since the HPIV1 C proteins specifically interacted with the Bro1 domain of Alix in our experiments, we expected to see an effect on viral replication or budding after knocking down Alix expression. However, knocking down the expression of Alix by RNA interference had no effect on HPIV1 replication or budding (**Fig. 6** **and Fig. S2**). This is in agreement with the observation that Alix knock-down also does not affect budding of the murine virus SeV [23]. For SeV, the budding of virus-like particles (VLPs) was reported to be reduced 5-fold when a dominant-negative (DN) vacuolar protein sorting-associated protein 4 (VPS4) was over-expressed [24]. VPS4 is an ATPase that interacts with ESCRT-III to catalyze the disassembly of the ESCRT complex and recycle its components [44]. An examination of the data showed that over-expression of DN-VPS4 also reduced the budding of VLPs by 5-fold even in the absence of C protein expression, suggesting that VLP budding utilizes the ESCRT system but that the C proteins may not be essential to this process. However, it was later reported that neither Alix knock-down nor DN-VPS4 over-expression affected SeV budding at all during an active infection [23]. Therefore, whether Alix promotes budding by SeV remains controversial. Importantly, we note that the lack of an effect of Alix knock-down on HPIV1 replication or budding might be due to the large redundancy that exists among host cell Bro1-containing proteins. For example, other Bro1 domain-containing proteins, including Brox, HD-PTP, and the rhophilins, have been shown to bind the HIV nucleocapsid, bind Chmp4b, and stimulate virus-like particle production of both HIV and hepatitis C virus [45], [46], [47]. Thus, the finding that the replication and budding of HPIV1 was unaffected by Alix knock-down may simply reflect the presence of other Bro1-containing proteins that compensate for the reduction in Alix.

To determine whether the ESCRT pathway might still play an important role in HPIV1 replication, we decided to test whether Chmp4 knock-down yielded a detectable phenotype during HPIV1 infection since the C proteins and Chmp4b bind at the same site in the Bro1 domain of Alix. Notably, redundancy also exists within the family of Chmp4 proteins. Three isoforms exist that can each bind to the Bro1 domain of Alix, namely Chmp4a, b, and c, with Chmp4b being the primary binding partner of Alix [37]. Because of this redundancy, we needed to knock-down all three isoforms simultaneously. We found that knocking down the expression of Chmp4 led to a 100-fold reduction in WT HPIV1 titer (**Fig. 7A**). This reduction in viral titer was paralleled by a decrease in the release of viral N protein into the cell culture media even though the amount of intracellular N protein was similar, indicative of an effect on the release of viral particles (**Fig. 7C**). Conversely, over-expression of Chmp4b resulted in an increase in the amount of viral N protein released extracellularly. Co-expression of Alix with Chmp4b blocked this increase, presumably because the intracellular environment was overloaded with Alix that preferentially bound Chmp4b instead of the HPIV1 C proteins (**Fig. 7D**). Consistent with this premise, we found that Alix does bind Chmp4b much more avidly than it binds the HPIV1 C proteins, and Chmp4b is capable of out-competing C proteins for the interaction with Alix (**Fig. 8**).

We noted that, in single-step growth experiments, the replication of P(C-) HPIV1, a mutant virus that does not express any of the C proteins, was approximately 100-fold less than that of WT HPIV1 (**Fig. 7A**) [10], [11]. Knocking down the expression of Chmp4 in the context of infection with P(C-) HPIV1 resulted in only a small additional reduction in viral replication. In contrast, knocking down the expression of Chmp4 in the context of infection with WT HPIV1 reduced viral replication to a much greater extent. The similarity of the effect (approximately 100-fold reduction in viral titers) of knocking down Chmp4 expression versus knocking out C protein expression is consistent with the idea that both Chmp4 and the viral C proteins play a significant, albeit non-essential, role in virus budding. Notably, a decrease in P(C-) titers was also observed with Chmp4 knock-down, but the effect was relatively small, ranging from 0.5 to 1 log_10_. The decrease in the amount of viral nucleocapsid released into the cell culture media during P(C-) infection compared to WT infection of Chmp4 knock-down cells was also relatively small (**Fig. 7A and C**). Thus, knocking down Chmp4b expression may have a small effect on viral budding independent of the C proteins, supporting the notion that the C proteins play an important role in enhancing viral budding but are not fully essential to this process. This is offered with the caveat that the absence of the C proteins can have other effects that could contribute to reduced replication of the P(C-) virus, including increased apoptosis and increased IFN production and signaling. However, these effects should be less important in the budding experiments, such as in **Fig. 7A**, because of their relatively late onset [10], [11].

Chmp4 expression might also have an effect on the expression level of Alix. We noted that the expression of the C proteins did not significantly alter the levels of Alix. For instance, in Figures 1D and E, 3B, and 5J, the expression of the HPIV1 C proteins did not increase the expression level of Alix. However, the over-expression of Chmp4, independent of the presence of the C proteins, appeared to reduce the expression level of Alix (Fig. 7D and 8). The mechanism by which this occurs remains to be determined but may involve increased Alix turnover.

The roles of Alix, Chmp4b, and the viral C proteins in HPIV1 infection remain incompletely understood, but a preliminary model can be proposed. We suggest that Alix (and potentially other Bro1-containing proteins) binds the C proteins during HPIV1 infection and serves to recruit C to the cytoplasmic face of late endosomes. We have already noted that this might account for the aggregation of C and STAT1 complexes to this region [17]. In addition, we (unpublished data) and others have observed an association between the HPIV1 and SeV C proteins and proteins of the nucleocapsid/polymerase complex, including the N and L proteins [48]. Chmp4 might then recruit the Alix-C-nucleocapsid and/or Alix-C-STAT1 complexes to a common site on the cytoplasmic face of late endosomes which serves as the origin for virus assembly and budding and also for interference with host IFN signaling and production. During protein trafficking, there is a constant cycling of protein between the membrane and cytoplasm, leading to both free cytoplasmic and membrane-bound pools of protein [49], [50]. It is possible that Alix undergoes a similar exchange process, whereby both free and membrane-bound Alix is concentrated at the late endosome, possibly via its ability to interact with Chmp4. The free cytoplasmic Alix is available to bind C and similarly concentrate C in the vicinity of the late endosome. Chmp4, which preferentially binds Alix, might then displace the nucleocapsids into the lumen of the nascent bud and drive neck scission. The HPIV1 virus particle within the intraluminal vesicle can now bud from infected cells upon fusion of the endosomal membrane with the plasma membrane. HIV can likewise bud into intracellular vesicles, particularly in macrophages. HIV-containing endosomes have been visualized by electron microscopy that fuse with the plasma membrane and release virus particles that carry several MVB markers [51]. This same process might also drive HPIV1 budding from the plasma membrane. In conclusion, the C proteins of HPIV1 allow the virus to interact with the host cell’s ESCRT pathway, and, although not essential for viral replication, this plays an important and complex role in the overall viral life cycle. Interfering with this interaction, such as through mutation or deletion of the C proteins, may be an important mechanism of attenuation and is represented in a live-attenuated HPIV1 vaccine presently in clinical trials.

## Supporting Information

Figure S1 **Intracellular co-localization of the C proteins with endogenous Alix and the late endosomal compartment during HPIV1 infection.** 293 T cells were mock-infected (**row 1**), infected with WT HPIV1 (**row 2**), infected with F170S HPIV1 (**row 3**), or infected with P(C-) HPIV1 (**row 4**) and after 48 h of incubation the cells were fixed, permeabilized, and analyzed by immunofluorescence with the antibodies indicated for each column. Nuclei were visualized with DAPI staining (blue). Both the WT and F170S C proteins (red) and Alix (green) co-localized (orange) in HPIV1-infected cells.
(TIF)

Figure S2 **Alix knock-down does not affect the growth of either HPIV1, HPIV2, or SeV.** Stable A549-derived cell lines were generated in which Alix expression was constitutively knocked-down (kd) using shRNA. 2 cell lines using 2 different shRNA constructs were used to ensure reproducibility of the data. These cells were infected with HPIV1 (**A**), HPIV2 (**B**), or SeV (**C**) at a MOI of 0.01, and the supernatants were sampled every other day up to day 5 post-infection to determine viral titers by limiting dilution analysis. The y-axis is in a log_10_ scale. **D.** The knock-down of Alix expression in the A549-derived cell lines was confirmed by Western blotting.
(TIF)
