# Supplementary figures and images for: Alix Serves as an Adaptor That Allows Human Parainfluenza Virus Type 1 to Interact with the Host Cell ESCRT System
Source: PLoS One. 2013 Mar 19;8(3):e59462. doi: 10.1371/journal.pone.0059462 (PMC3602193; doi:10.1371/journal.pone.0059462)

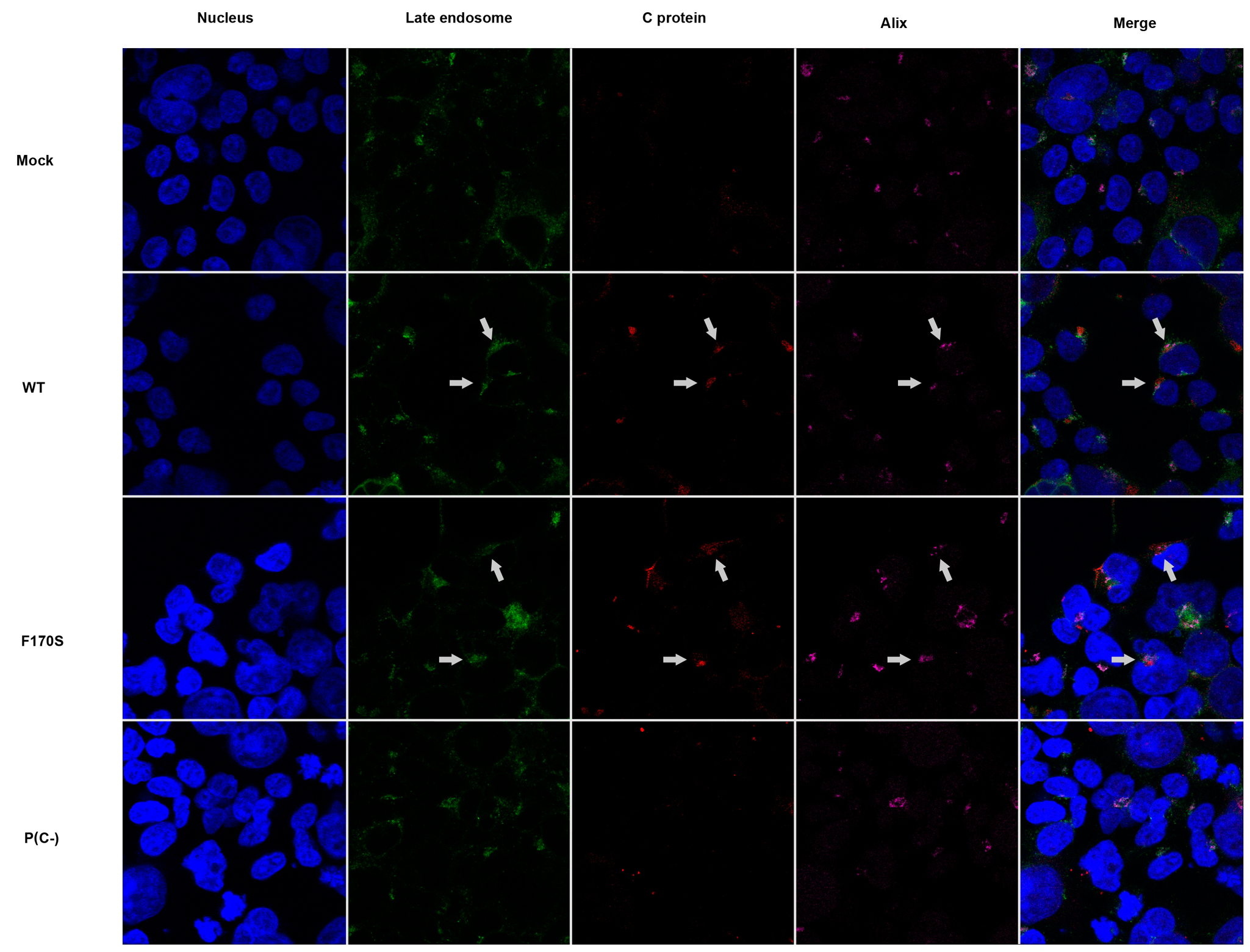

Supplement: Figure S1 — Intracellular co-localization of the C proteins with endogenous Alix and the late endosomal compartment during HPIV1 infection. 293 T cells were mock-infected (row 1), infected with WT HPIV1 (row 2), infected with F170S HPIV1 (row 3), or infected with P(C-) HPIV1 (row 4) and after 48 h of incubation the cells were fixed, permeabilized, and analyzed by immunofluorescence with the antibodies indicated for each column. Nuclei were visualized with DAPI staining (blue). Both the WT and F170S C proteins (red) and Alix (green) co-localized (orange) in HPIV1-infected cells. (TIF) [file pone.0059462.s001.tif]

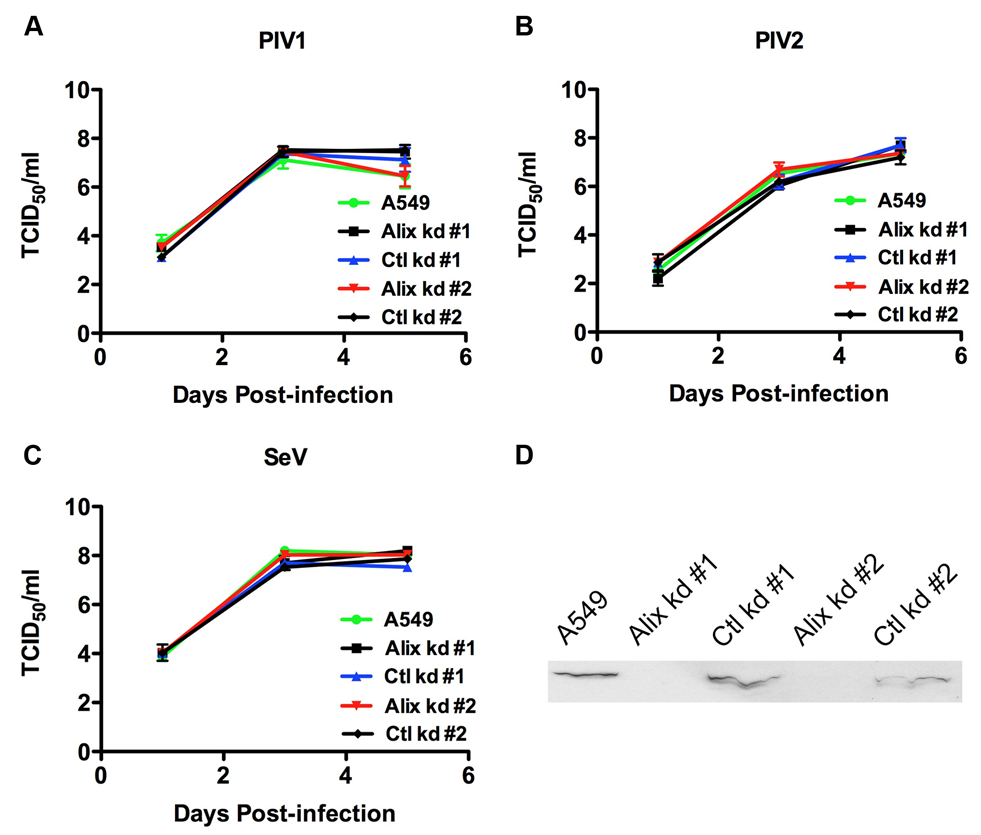

Supplement: Figure S2 — Alix knock-down does not affect the growth of either HPIV1, HPIV2, or SeV. Stable A549-derived cell lines were generated in which Alix expression was constitutively knocked-down (kd) using shRNA. 2 cell lines using 2 different shRNA constructs were used to ensure reproducibility of the data. These cells were infected with HPIV1 (A), HPIV2 (B), or SeV (C) at a MOI of 0.01, and the supernatants were sampled every other day up to day 5 post-infection to determine viral titers by limiting dilution analysis. The y-axis is in a log10 scale. D. The knock-down of Alix expression in the A549-derived cell lines was confirmed by Western blotting. (TIF) [file pone.0059462.s002.tif]
